# Initiation, extension, and termination of RNA synthesis by a paramyxovirus polymerase

**DOI:** 10.1371/journal.ppat.1006889

**Published:** 2018-02-09

**Authors:** Paul C. Jordan, Cheng Liu, Pauline Raynaud, Michael K. Lo, Christina F. Spiropoulou, Julian A. Symons, Leo Beigelman, Jerome Deval

**Affiliations:** 1 Alios BioPharma, Inc. a Janssen Pharmaceutical Company of Johnson & Johnson, South San Francisco, California, United States of America; 2 Centers for Disease Control and Prevention, Atlanta, Georgia, United States of America; Institut Pasteur, FRANCE

## Abstract

Paramyxoviruses represent a family of RNA viruses causing significant human diseases. These include measles virus, the most infectious virus ever reported, in addition to parainfluenza virus, and other emerging viruses. Paramyxoviruses likely share common replication machinery but their mechanisms of RNA biosynthesis activities and details of their complex polymerase structures are unknown. Mechanistic and functional details of a paramyxovirus polymerase would have sweeping implications for understanding RNA virus replication and for the development of new antiviral medicines. To study paramyxovirus polymerase structure and function, we expressed an active recombinant Nipah virus (NiV) polymerase complex assembled from the multifunctional NiV L protein bound to its phosphoprotein cofactor. NiV is an emerging highly pathogenic virus that causes severe encephalitis and has been declared a global public health concern due to its high mortality rate. Using negative-stain electron microscopy, we demonstrated NiV polymerase forms ring-like particles resembling related RNA polymerases. We identified conserved sequence elements driving recognition of the 3′-terminal genomic promoter by NiV polymerase, and leading to initiation of RNA synthesis, primer extension, and transition to elongation mode. Polyadenylation resulting from NiV polymerase stuttering provides a mechanistic basis for transcription termination. It also suggests a divergent adaptation in promoter recognition between pneumo- and paramyxoviruses. The lack of available antiviral therapy for NiV prompted us to identify the triphosphate forms of R1479 and GS-5734, two clinically relevant nucleotide analogs, as substrates and inhibitors of NiV polymerase activity by delayed chain termination. Overall, these findings provide low-resolution structural details and the mechanism of an RNA polymerase from a previously uncharacterized virus family. This work illustrates important functional differences yet remarkable similarities between the polymerases of nonsegmented negative-strand RNA viruses.

## Author summary

RNA viruses replicate and transcribe their genomes using complex enzymatic machines known as RNA-dependent RNA polymerases. The chemical reactions driving nucleotide addition are shared among nucleic acid polymerases but the underlying mechanisms of RNA biosynthesis and the complex polymerase structures are diverse. Of these RNA viruses is the paramyxovirus family, which includes major human pathogens. Paramyxoviruses have common biological and genetic properties but little is known about their replication machinery. Insights into the structure, function, and mechanisms of RNA synthesis of one paramyxovirus polymerase will likely extend to the entire virus family. An emerging, highly pathogenic paramyxovirus is Nipah virus (NiV), which causes encephalitis in humans. We have purified NiV polymerase, probed its enzymatic and biophysical properties and developed it as a model paramyxovirus polymerase. We investigated template strand sequence elements driving RNA biosynthesis for NiV polymerase and obtained a snapshot of NiV polymerase molecular organization using electron microscopy to provide the first structural information on a paramyxovirus polymerase. This work extends previous knowledge by producing the first recombinant paramyxovirus polymerase and using this protein in enzymatic assays to highlight key functional and structural characteristics for the design of new medicines.

## Introduction

Paramyxoviruses describe a family of viruses responsible for significant disease, ranging from lower respiratory disease due to parainfluenza virus and measles virus as well as emerging pathogens such as Nipah and Hendra viruses causing severe encephalitis. The replication machinery of these viruses, the polymerase complex, is largely unstudied due to their complexity and size and to date there has been no report of a recombinant paramyxovirus polymerase. The biochemistry and importantly, the potential inhibition of these protein complexes represents a significant opportunity for understanding and preventing disease.

Nipah virus (NiV) is an emerging pathogenic paramyxovirus part of the *Henipavirus* genus within the *Paramyxoviridae* family [1,2]. NiV infection in humans is characterized by systemic vasculitis, ultimately resulting in fatal encephalitis [3,4]. Due to its highly pathogenic nature and the lack of approved therapeutics or vaccines, NiV has been classified as a category C priority pathogen by the Centers for Disease Control and Prevention and the National Institute of Allergy and Infectious Diseases [5]. There are no approved antiviral drugs or vaccines for NiV infection [6].

NiV has a nonsegmented negative-strand (NNS) RNA genome of approximatively 18 kilobases [7]. It contains the classic non-coding regions such as the 3′ leader (*Le*) and 5′ trailer (*Tr*) sequences, gene-start (GS), gene-end (GE), RNA-editing, and intergenic sequences in addition to coding regions for six proteins: nucleoprotein (N), large (L) protein, phosphoprotein (P), matrix (M) protein, fusion (F) protein, and glycoprotein (G) [7–10]. The genomes of NNS RNA viruses are transcribed and replicated by the RNA-dependent RNA polymerase (RdRp). The RdRp is a multiprotein complex composed of the L protein and the P protein [11]. The *Le* region is a bipartite promoter that is highly conserved among Paramyxoviruses [12]. In NiV, mutations within the two conserved promoter elements (nt 1–12 and 79–91) result in loss of minigenome function [13].

The L protein of the NNS RNA viruses serves the two main enzymatic functions responsible for RdRp and mRNA cap formation. The L protein ranges in size within NSS viruses but is approximately 250 kDa [11]. Six highly conserved amino acid sequences, known as conserved regions (CR) I to VI, are found in the L protein of all NNS RNA viruses [14,15]. The L protein exists in a complex with the P protein, which acts as a molecular chaperone for the polymerase and enhances processivity [16]. Structural characterization of the L-P complex remains challenging due to the large size of the L protein and the difficulty of obtaining adequate quantities of highly purified proteins. However, the cryo-electron microscopy (EM) structure of the L protein from vesicular stomatitis virus (VSV), a negative-sense RNA virus of the *Rhabdoviridae* family, has recently revealed how the domain organization and enzymatic function are organized in a three-dimensional structure [17]. Negative-stain EM of VSV L protein showed the RdRp domain containing the CRIII adopts a ring- or doughnutlike architecture decorated with multiple appendages in various orientations [18]. CRIV and V, mapped to the capping domain, appear as a single, globular appendage covering the hole of the doughnut. Additionally, there are three small, globular appendages corresponding to the connector domain, the methyl-transferase domain, and the C-terminal domain (CTD). This CTD was described as the +domain in the structure of a HMPV L protein fragment [19]. The +domain is critical for HMPV MTase activity. The addition of VSV P protein causes a molecular rearrangement that results in the appendages forming a condensed tail on one side of the core structure.

Conserved sequences within the RdRp and capping domains suggest that the molecular organization of L proteins from other viruses may be similar. The X-ray crystal structure of the NiV P multimerization domain and the N protein have recently been solved [20,21]. The P protein acts as a link between the L protein and the nucleocapsid template, and acts as a chaperone for an RNA-free form of the N protein [20]. The NiV P protein is composed of three domains, known as the N-terminal domain, the P multimerization domain, and the X domain. The P multimerization domain has been reported as a tetrameric coiled coil while others have reported it forms an elongated coiled-coil trimer[20,22]. The structure of P in relationship to the L protein is not known. The P protein may undergo structural re-organization in the context of the L-P or L-P-N complexes. Details of this structural re-organization, combined with the known structure of the NiV N protein, will provide insights into the NiV enzymatic machinery [21]. It is thought that NiV employs the same or similar mechanism as other NNS RNA viruses such as VSV (*Rhabdoviridae*) and respiratory syncytial virus (RSV; *Pneumoviridae*). Current mechanistic knowledge of paramyxoviral L proteins has been limited to bioinformatic studies. There has been no report of the purification or biochemical characterization of an active paramyxoviral polymerase, which presents an exciting opportunity to probe the enzymatic properties of the polymerase of an emerging pathogen.

Here, we present the first report of the expression, purification and biophysical and biochemical characterization of a paramyxovirus polymerase complex. Visualization of purified NiV polymerase by negative stain-EM shows ring-like particles. Using a combination of enzymatic approaches, we demonstrate that NiV polymerase is active in *de novo* and primer-dependent RNA synthesis. We identified sequence requirements for promoter recognition and for polyadenylation by NiV polymerase. We discovered that NiV polymerase is much more prone to stuttering than RSV polymerase, which could explain differences in promoter sequences between pneumo- and paramyxoviruses. Considering NiV’s status as a priority pathogen, we also characterized clinically-relevant nucleotide analogs that inhibit NiV replication by a mechanism of delayed chain termination. The purified enzyme represents a powerful tool for understanding the enzymatic function of the polymerase from a previously uncharacterized family of viruses and has significant implications for the development of novel inhibitors of NiV and other related viruses.

## Results

### Expression, purification, and molecular organization of NiV L-P polymerase complex

**Fig 1:**
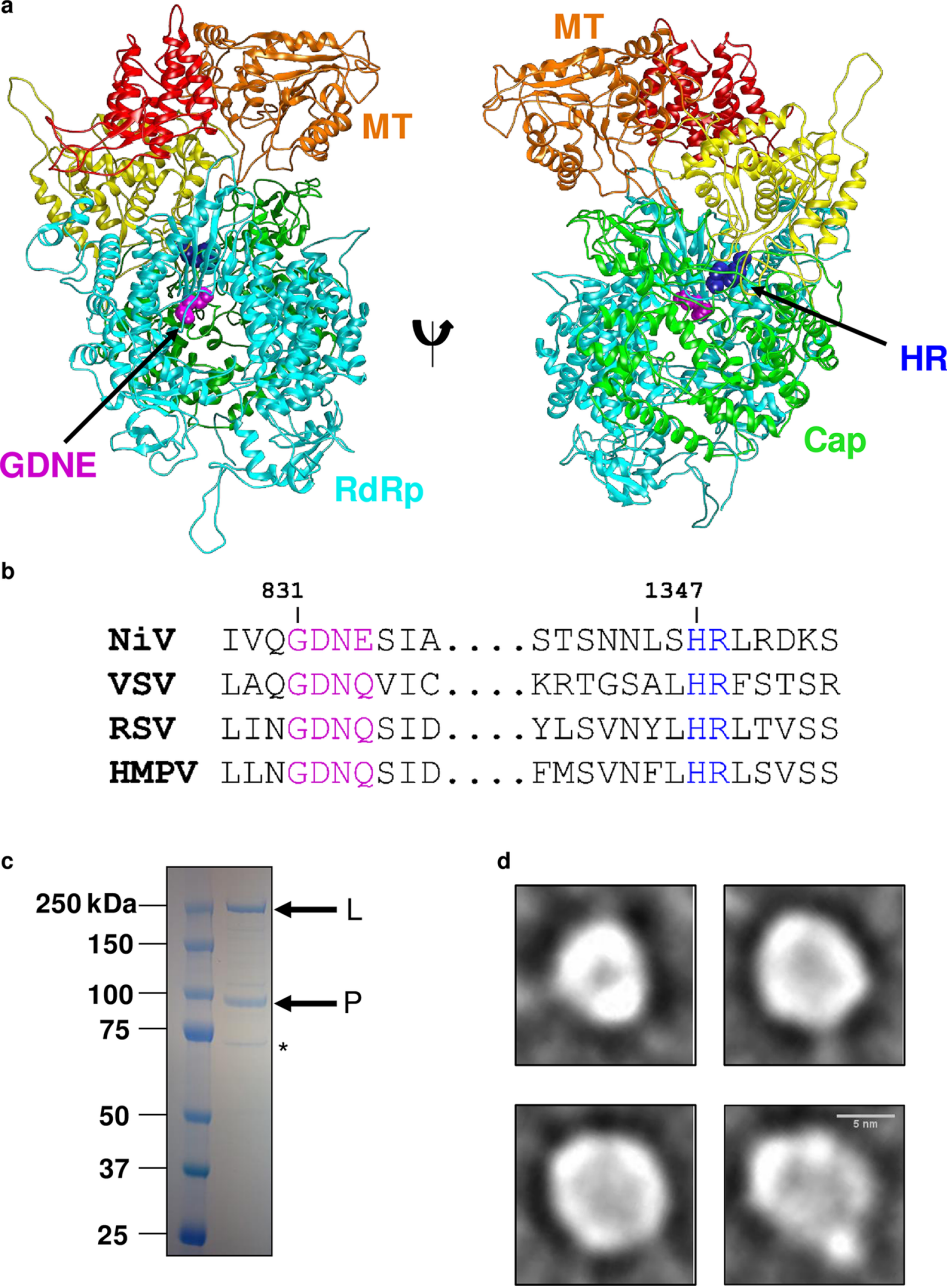
Expression, purification, and molecular organization of NiV polymerase complex. (a) A homology-based three-dimensional structure of the NiV polymerase L protein highlighting the RdRp domain, the methyltransferase (MT), and capping domain. The NiV L protein contains conserved residues (GDNE and HR motifs), corresponding to catalytically active sites within the protein that are in close spatial proximity. (b) Sequence alignment of the L proteins for NiV, vesicular stomatitis virus (VSV), respiratory syncytial virus (RSV), and human metapneumovirus (HMPV) shows good conservation of GDN and HR motifs. (c) SDS-PAGE analysis of recombinant NiV L-P proteins with asterisk indicating HSC70 protein. (d) Four representative class averages of the NiV polymerase complex imaged using negative stain EM. Scale bar (5 nm) is shown in bottom right micrograph.

The paramyxoviral polymerases comprise a large (L) protein and a phosphoprotein (P). We hypothesized that the NiV L protein adopts a structure similar to the VSV L protein given their moderate sequence similarity (35%). Using homology modeling to generate a three-dimensional view of the NiV L protein, our model indicates the RdRp domain adopts a classic right-handed configuration (Fig 1A). The sequences of NNS RNA virus L proteins contain conserved residues that are in close spatial proximity to one another and correspond to catalytically active sites within the protein. The NiV L protein contains a 831-GDNE-834 motif within the RdRp domain in contrast to the GDNQ sequence in most other NNS RNA virus L proteins (Fig 1B). In addition, highly conserved histidine and arginine residues are found within the capping domain. The histidine of the HR motif forms a covalent intermediate during polyribonucleotidyltransferase (PRNTase) reaction in VSV polymerase, although this enzymatic activity has not been reported for NiV (Fig 1B). Importantly, the close spatial proximity of these conserved catalytic sites suggests a potential functional cooperativity among multiple L protein domains.

We then produced NiV polymerase through the co-expression of the N-terminal FLAG-tag L and P proteins of NiV within insect cells infected with a single recombinant baculovirus clone. The protein was purified by FLAG-tag and size exclusion-chromatography (SEC). Fractions corresponding to a peak near the void volume were pooled, concentrated, and analyzed by SDS-PAGE revealing bands consistent with the expected molecular weights of the L protein (260 kDa) and P protein (80 kDa) (Figs 1C and S1). In order to understand and compare their effects in enzymatic assays, we expressed and purified NiV L(D832A-N833A)-P and NiV L(H1347A)-P containing mutations in the catalytic sites of the RdRp and capping domains, respectively. Fractions from a predominant peak were pooled for each protein, concentrated, and analyzed by SDS-PAGE to reveal bands consistent with the expected molecular weights of the L and P proteins (S2 Fig). The L proteins from NiV L(wild-type [wt])-P, NiV L(H1347A)-P, and NiV L(D832A-N833A)-P were excised from a denaturing gel and subjected to proteolysis followed by liquid chromatograph-tandem mass spectroscopy (LC-MS/MS) to verify the sequences were complete and to confirm amino acid substitutions. Results from these assays showed ≥95% sequence coverage for all three L proteins (example coverage map shown in S3 Fig). Similar results were obtained for the NiV P protein (coverage map in S3 Fig). A minor host contaminant protein co-purified with the NiV L-P complex (Fig 1C). This protein was also excised from a denaturing gel and positively identified using LC-MS/MS as a heat shock cognate (HSC) 70 protein. Previous work identified that heat shock protein 70 and/or HSC70 co-purifies with RSV polymerase, a closely related polymerase to NiV polymerase [23]. Typical protein concentrations ranged from 0.7–1.7 mg/mL, with an average yield of about 0.5 mg per liter of insect cell culture.

We probed the molecular architecture of the NiV L(wt)-P complex through visualization by negative-stain transmission EM. The images revealed small, 5–10 nm globular particles and 20–70 nm larger particles that appeared to be clumped or aggregated protein (S1 Fig). The smaller particles were selected for alignment and two-dimensional (2D) classification. Class averages showed both smaller, 5–8 nm globular particles with even density (Fig 1D, top left) and larger 8–11-nm globular or ring-like particles (Fig 1D, top right and bottom row) that appear to have an indentation or cavity in the center. Our data do not show globular appendages that could map to other regions of the L or P proteins, suggesting the NiV L(wt)-P complex adopts a unique molecular arrangement. Using these insights, we explored the activity of the protein in functional enzymatic assays.

### *De novo* RNA synthesis by NiV L-P

**Fig 2:**
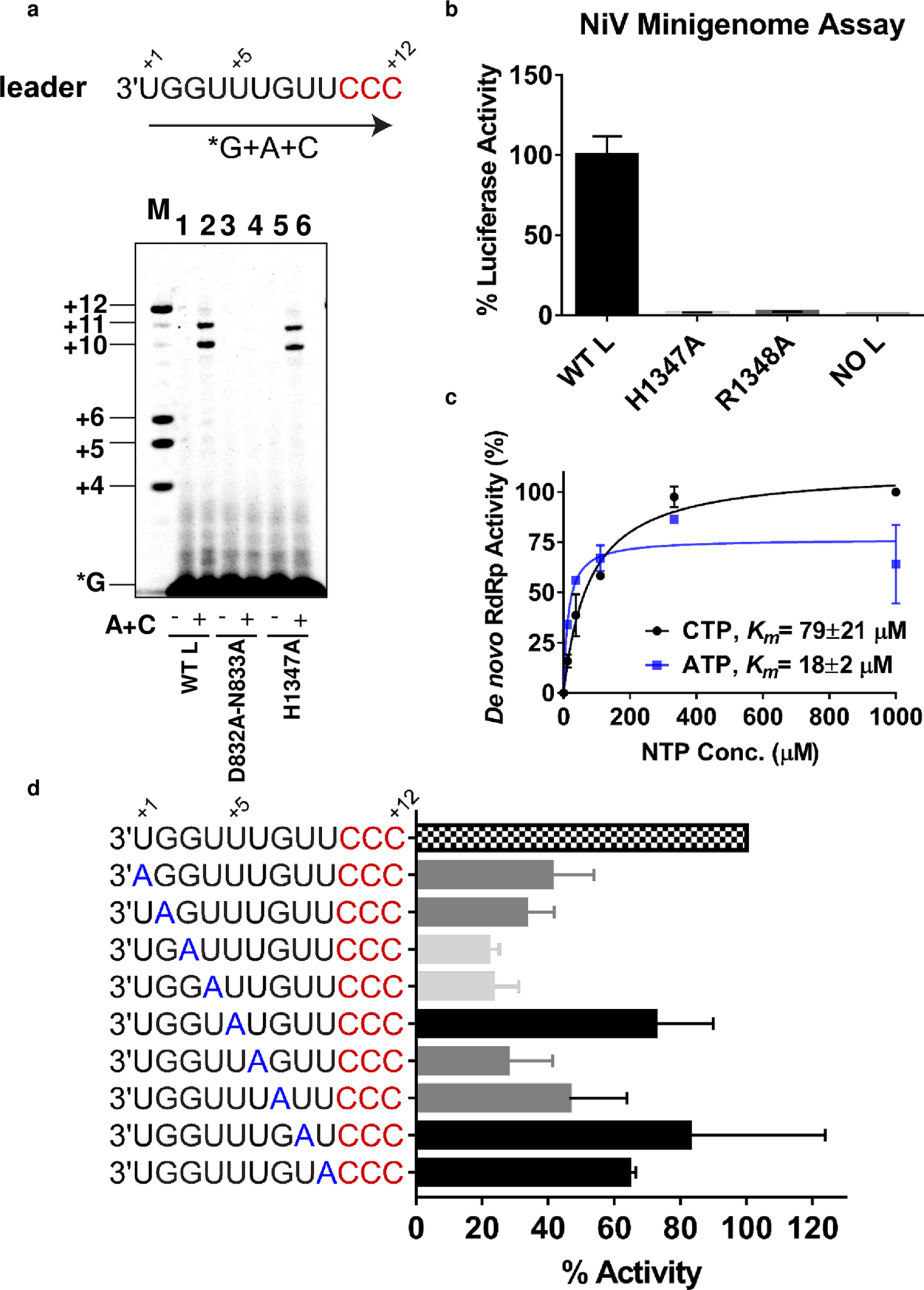
*De novo* RNA synthesis by NiV L-P and analysis of promoter sequence requirements. (a) The 12-mer RNA template from the leader promoter region of the NiV genome was used in a *de novo* RdRp assay and products were analyzed using a 22.5% polyacrylamide urea sequencing gel. NiV L(wild-type [wt])-P, abbreviated as WT L, and template were incubated in the presence of α^33^P-GTP tracer (lane 1) or with the addition of ATP (A) and CTP (C) (lane 2). NiV L(D832A-N833A)-P mutant, abbreviated as D832A-N833A, and template were incubated in the presence of α^33^P-GTP tracer (lane 3) or with the addition of ATP and CTP (lane 4). NiV L(H1347A)-P, abbreviated as H1347A, and template were incubated in the presence of α^33^P-GTP tracer (lane 5) or with the addition of ATP and CTP (lane 6). Product sizes are indicated using a kinase-labeled set of four combined oligonucleotides: 4-, 5-, 6-, and 12-mer primer (lane M). (b) A nanoluciferase-based NiV minigenome assay comparing luciferase activity for NiV L(wt), NiV L(H1347A), NiV L(R1348A), and a control sample with no L. Sample size was n = 2, error bars represent standard deviation, and sample luciferase activity was normalized to NiV L(wt). (c) Product formation was quantified and the Michaelis constant (*K*_*m*_) for ATP and CTP was calculated. Sample size was n = 2 and error bars represent standard deviation. (d) Using systematically introduced adenine at the first nine bases of the promoter sequence, RNA synthesis activity was quantified, and normalized to the 12-mer RNA template from the leader promoter. For these experiments, nucleotide substrates included ATP+ CTP+UTP at 1 mM each, and α^33^P-GTP. Samples size was n = 2 and error bars represent standard deviation.

The RdRp activity of NiV L(wt)-P was measured in a radiometric assay using α^33^P-labeled guanosine triphosphate (GTP) as nucleotide substrate for the reaction. The leader (*Le*) promoter region at the 3′-end of the NiV genome was chosen to design the 12-mer synthetic RNA template for the RdRp reaction because it contains the authentic sequence recognized by the polymerase during virus replication (Fig 2A). This uridine-rich template contains three consecutive cytidines at its 5′-terminus that are required for product detection through α^33^P-GTP incorporation into the nascent complementary RNA strand. In the presence of NiV L(wt)-P, α^33^P-GTP was not sufficient to promote RNA synthesis without other nucleotides (Fig 2A, lane 1). The addition of cytosine triphosphate (CTP) + adenosine triphosphate (ATP) was required for the formation of 10- and 11-mer products (Fig 2A, lane 2). In the presence of the NiV L(D832A-N833A)-P mutant, nearly 100% of product formation was inhibited (Fig 2A, lanes 3 and 4). In comparison, the H1347A mutation resulted in only ~50% reduction in *de novo* RNA synthesis relative to the wild-type enzyme (Fig 2A, lanes 5 and 6). In the cell-based minigenome assay, the adjacent H1347A and R1348A mutations completely blocked the luciferase reporter activity (Fig 2B). The more pronounced detrimental effect of H1347A in the minigenome assay compared with the RdRp assay is most likely due to RNA capping impairment in cells. In the *de novo* RdRp assay, ATP and CTP were both required to support RNA synthesis (S4 Fig), with a *K*_*m*_ value for CTP and ATP of 79±21 μM and 18±2 μM, respectively (Figs 2C and S4).

### Promoter sequence requirements for *de novo* RNA synthesis

The *Le* bipartite promoter region is highly conserved among Paramyxoviruses [12]. In NiV, its first element (nt 1–12) contains a uridine-rich sequence also found in other NNS RNA viruses. In the NiV L-P enzymatic assay, swapping the RNA template from NiV to RSV-derived promoter resulted in comparable RdRp activity (S5 Fig). To better understand the specificity of recognition of the NiV Le region nt 1–12, we systematically introduced an adenine at each of the first nine bases on the promoter sequence. Positions 5, 8, and 9 were minimally affected by changes in promoter sequence (Fig 2D, black bars). An adenine at position 1, 2, 6, or 7 resulted in a 50–75% reduction in RNA synthesis (Fig 2D, dark gray bars). Positions 3 and 4 were not only the most sensitive to nucleobase change with ~80% loss in activity (Fig 2D, light gray bars), changes at these positions also resulted in unexpected RNA product sizes that were both shorter and longer than the reference promoter sequence (S6 Fig). The longer aberrant products might result from enzyme stuttering. Taken together, the result of this adenine scan indicates that nucleotides 3 and 4 on the Le region are the most important for promoter recognition by NiV L-P.

### Primer extension and inhibition of RNA synthesis

**Fig 3:**
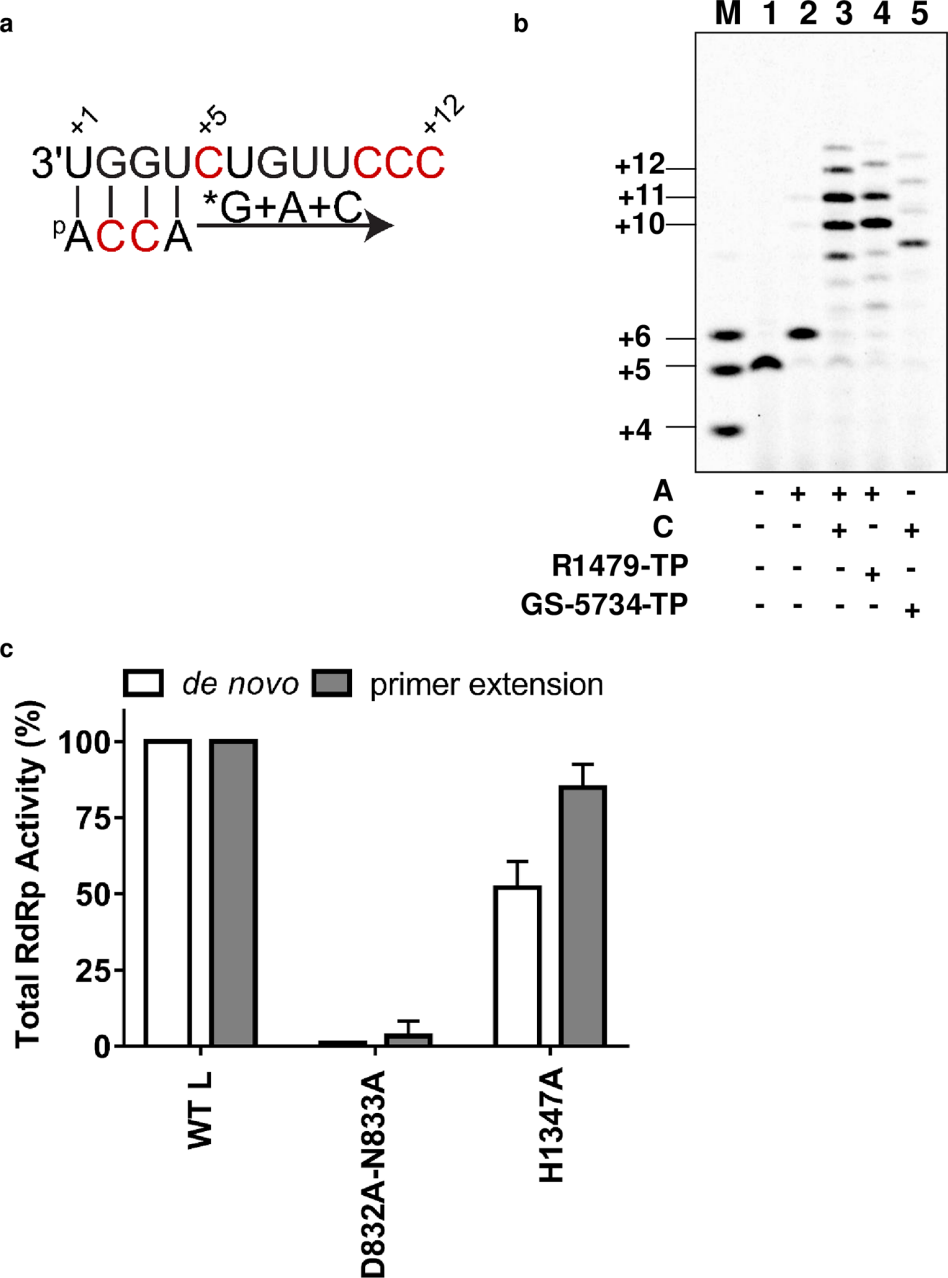
Primer-dependent RNA synthesis and inhibition by NiV L-P. (a) A 12-mer template with four bases complementary to a short 4-nucleotide primer was used to analyze primer-extension activity of NiV L(wt)-P. (b) Product sizes are indicated using a 4-5-6-mer combined RNA size marker (lane M). NiV L(wt)-P, template, and primer were incubated in the presence of α^33^P-GTP tracer (lane 1), α^33^P-GTP + ATP (lane 2), α^33^P-GTP + ATP + CTP (lane 3), α^33^P-GTP + ATP + R1479-TP (lane 4), and α^33^P-GTP + CTP + GS-5734-TP (lane 5). (c) Total RdRp activity in primer extension and *de novo* formats reported after normalizing to the NiV L(wt)-P (see S7 Fig). Samples size was n = 2 and error bars represent standard deviation.

Elongation of RNA synthesis by NiV L-P was mimicked by using a short 4-nucleotide (nt) primer complementary to the 3′-end of the 12-mer *Le* template. Since the nucleotide sequence at position 5 on the RNA template is not important (Fig 2D), it was changed from uridine (U) to C to enable enzymatic primer labeling and 5-mer product formation by adding α^33^P-GTP to the NiV L(wt)-P RNA complex (Fig 3A, Fig 3B lane 1). The primer extension activity profile of NiV L(wt)-P was also compared with the RNA product from the D832A-N833A and H1347A mutants. As expected, the D832A-N833A mutation in the RdRp active site completely abrogated primer extension activity (Figs 3C and S7). In contrast, the H1347A mutation in the capping domain had little effect on primer extension compared with the wt enzyme (Fig 3C). In the primer extension assay, the 4-mer primer was fully converted by NiV L(wt)-P to 6-mer product in the presence of ATP (Fig 3B, lane 2). Compared with *de novo* RNA synthesis, the *K*_*m*_ requirement for ATP by NiV L(wt)-P was reduced by ~1,000-fold to 0.015±0.001μM (S8 Fig). The NiV RNA template only allows for one CTP addition event at position +7. Adding ATP + CTP to the reaction resulted in 10- and 11-mer products predominantly, with minor products at positions +9, +12, and +13 (Fig 3B, lane 3). R1479 is a cytidine analog recently found to inhibit NiV replication [24]. Replacing CTP with R1479 triphosphate (TP) did not inhibit 10- and 11-mer product formation (Fig 3B, lane 4). This indicates that in the NiV L-P assay, single incorporation of R1479-TP into the nascent RNA does not result in immediate chain termination. In comparison, the same reaction conducted with the obligate chain terminator 3′dCTP instead of CTP resulted in the expected 7-mer product (green arrow in S9 Fig). GS-5734, an adenosine analog, is also known to inhibit NiV replication [25]. Replacing ATP with GS-5734-TP inhibited 10- and 11-mer product formation, and resulted in accumulation of 9-mer product (Fig 3B, lane 5). This requires GS-5734-TP to be incorporated three times, at positions +6, +8, and +9. We conclude that GS-5734-TP is a delayed (or leaky) terminator of RNA chain synthesis, which is consistent with prior observations using RSV polymerase [26]. In comparison, use of 3′dATP instead of ATP resulted in the expected 6-mer RNA product (blue arrow in S9 Fig).

### The consensus GE sequence triggers polymerase slippage for NiV but not for RSV

**Fig 4:**
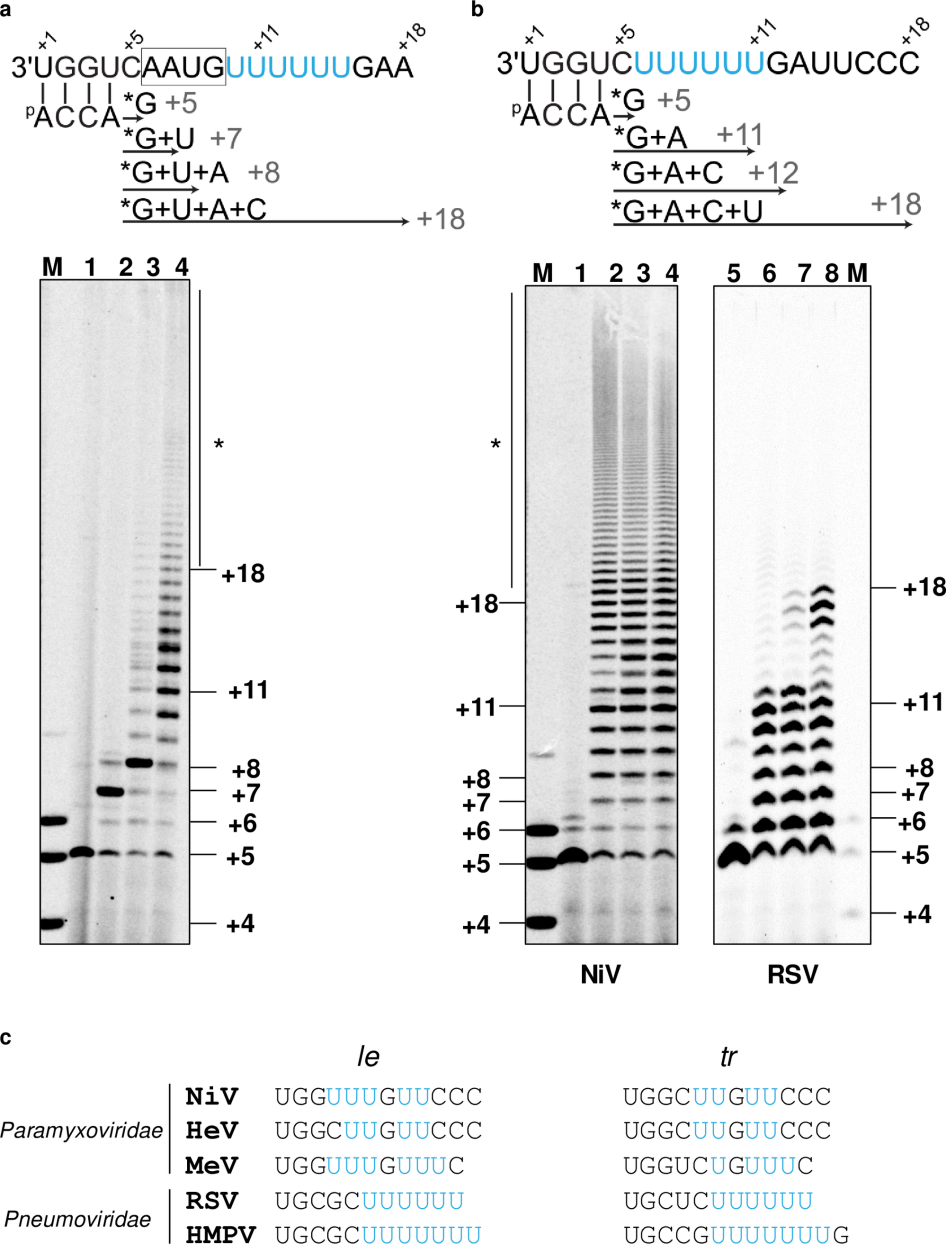
Analysis of polymerase slippage triggered by consensus GE sequence. (a) A designed template strand containing four bases complementary to a 4-mer primer followed by the NiV M-F GE signal. Product sizes are indicated using a 4-5-6-mer combined RNA size marker (lane M). NiV L(wt)-P, template and primer were incubated in the presence of α^33^P-GTP tracer (lane 1), α^33^P-GTP + UTP (lane 2), α^33^P-GTP + UTP + ATP (lane 3), or α^33^P-GTP + UTP + ATP + CTP (lane 4). (b) A poly(U)_6_ tract template was designed without the AAUG part of the GE signal to probe the effects of this upstream sequence on polyadenylation. Product sizes are indicated using a 4-5-6-mer combined RNA size marker (lane M). NiV L(wt)-P, template, and primer were incubated in the presence of α^33^P-GTP tracer (lane 1), α^33^P-GTP + ATP (lane 2), α^33^P-GTP + ATP + CTP (lane 3), or α^33^P-GTP + ATP + CTP + UTP (lane 4). RSV L-P, template, and primer were incubated in the presence of α^33^P-GTP (lane 5), α^33^P-GTP + ATP (lane 6), α^33^P-GTP + ATP + CTP (lane 7), or α^33^P-GTP + ATP + CTP + UTP (lane 8). Product sizes are indicated using a 4-5-6-mer combined RNA size marker (lane M). (c) Alignment of the 3′ leader (*le)* and 5′ trailer (*tr*) sequences Nipah virus (NiV), Hendra virus (HeV), Measles virus (MeV), respiratory syncytial virus (RSV), and human metapneumovirus (HMPV).

In paramyxoviruses and other NNS RNA viruses, the precise molecular mechanism for recognizing the non-coding GE signal as template for termination of gene transcription and polyadenylation is not well understood. Toward this aim, a new RNA template was designed to measure NiV L-P slippage leading to mRNA polyadenylation. This oligonucleotide sequence contains the first four bases complementary to the 4-mer primer, immediately followed with the M-F GE signal containing a AAUG block, followed by a poly(U)_6_ tract and a GAA intergenic region (Fig 4A). The 4-mer primer was converted into a 5-mer product with the addition of α^33^P-GTP (Fig 4A, lane 1). As expected, adding α^33^P-GTP + UTP resulted in predominantly a 7-mer product and adding α^33^P-GTP + UTP + ATP resulted in largely the 8-mer product (Fig 4A, lanes 2 and 3). With the addition of α^33^P-GTP + UTP + ATP + CTP, the polymerase produces longer RNA than the expected length (18 nt) indicative of polyadenylation (Fig 4A, lane 4). The polyadenylation results from the NiV polymerase stuttering along the poly(U) tract as part of the GE signal. A poly(U)_6_ tract template was designed without the AAUG part of the GE signal to understand the effects of this upstream sequence on polyadenylation. In this new template, the 4-mer primer was converted into a 5-mer product with the addition of α^33^P-GTP (Fig 4B, lane 1). Three other conditions were analyzed: α^33^P-GTP + ATP, α^33^P-GTP + ATP + CTP, and α^33^P-GTP + ATP + CTP + UTP. These three conditions yielded products significantly longer than expected, 11, 12, and 18 nt, respectively (Fig 4B, lanes 2, 3, and 4). Our results show that the sequence upstream of the poly(U) tract is not critical for polyadenylation and instead the efficiency of polyadenylation increases in the absence of the AAUG sequence. The same experiments were repeated with the RNA polymerase of RSV, a member of the *Pneumoviridae* family. This time, the four standard conditions (α^33^P-GTP alone, α^33^P-GTP + ATP, α^33^P-GTP + ATP + CTP, and α^33^P-GTP + ATP + CTP + UTP) yielded products mainly with the expected size of 5, 11, 12, and 18 nt, respectively (Fig 4B, lanes 5, 6, 7, and 8). Compared with NiV, the amount of polyadenylation resulting from RSV L-P stuttering was negligible. To confirm that RSV L-P is intrinsically less prone to polyadenylation, similar experiments were repeated with the authentic RSV *Le* promoter sequence containing a poly(U)_6_ tract. There again, RSV L-P generated products of the expected size, without any strong evidence of polyadenylation (S10 Fig). These results could explain why the genomic promoters in pneumovirus RNA contain a polyU tract that is not found in the corresponding paramyxovirus sequences (Fig 4C).

### Other factors regulating polyadenylation by NiV L-P

**Fig 5:**
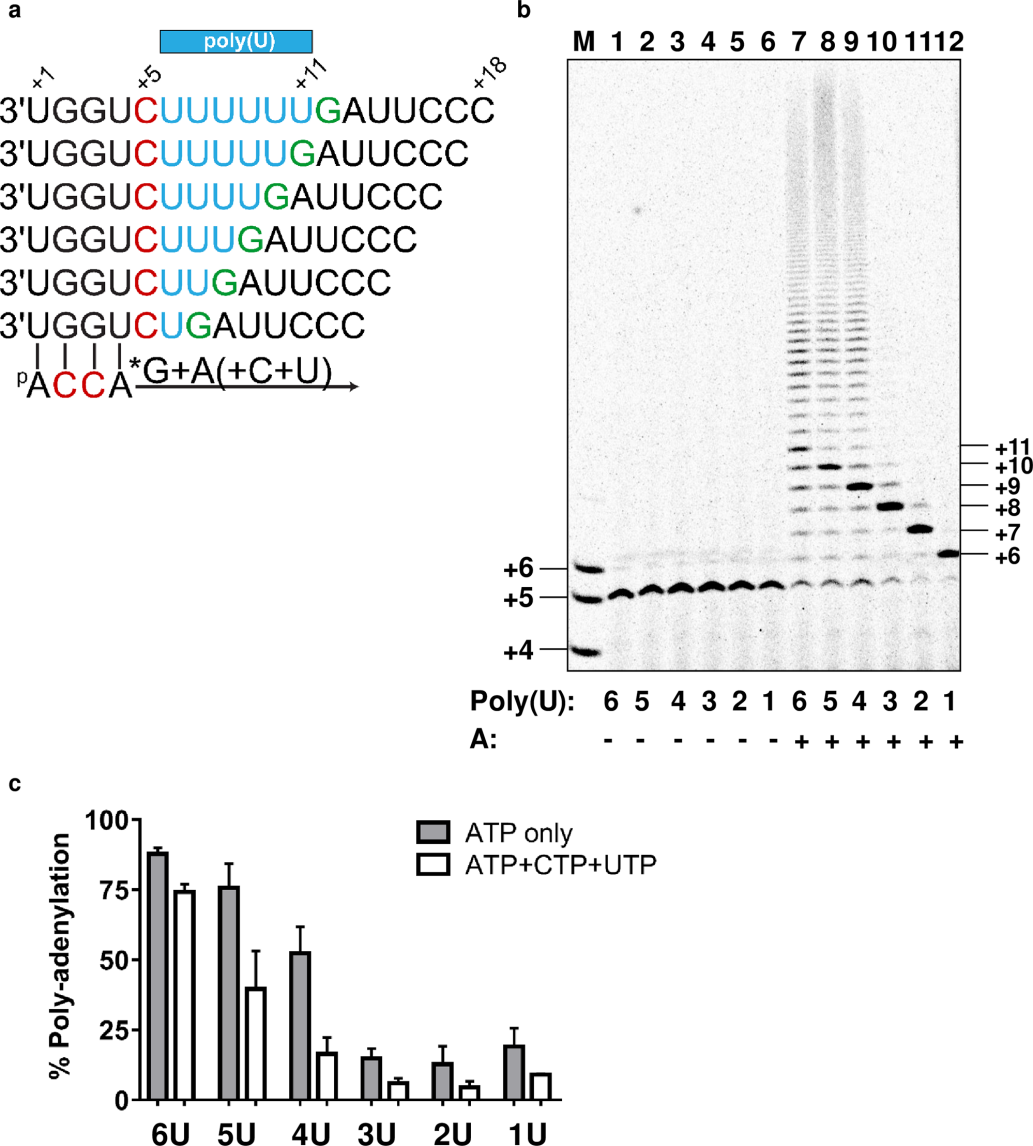
NiV polymerase polyadenylation. (a) RNA templates were designed with a variable number of uridines on the poly(U) template while maintaining the rest of the sequence unchanged. (b) Product sizes are indicated using a 4-5-6-mer combined RNA size marker (lane M). NiV L(wt)-P, template and primer were incubated in the presence of α^33^P-GTP (lanes 1–6), or α^33^P-GTP + ATP (lanes 7–12). (c) Percentage polyadenylation was quantitated for reactions containing α^33^P-GTP + ATP or α^33^P-GTP + ATP + CTP + UTP. Sample size was a minimum of n = 2 and error bars represent standard deviation.

**Fig 6:**
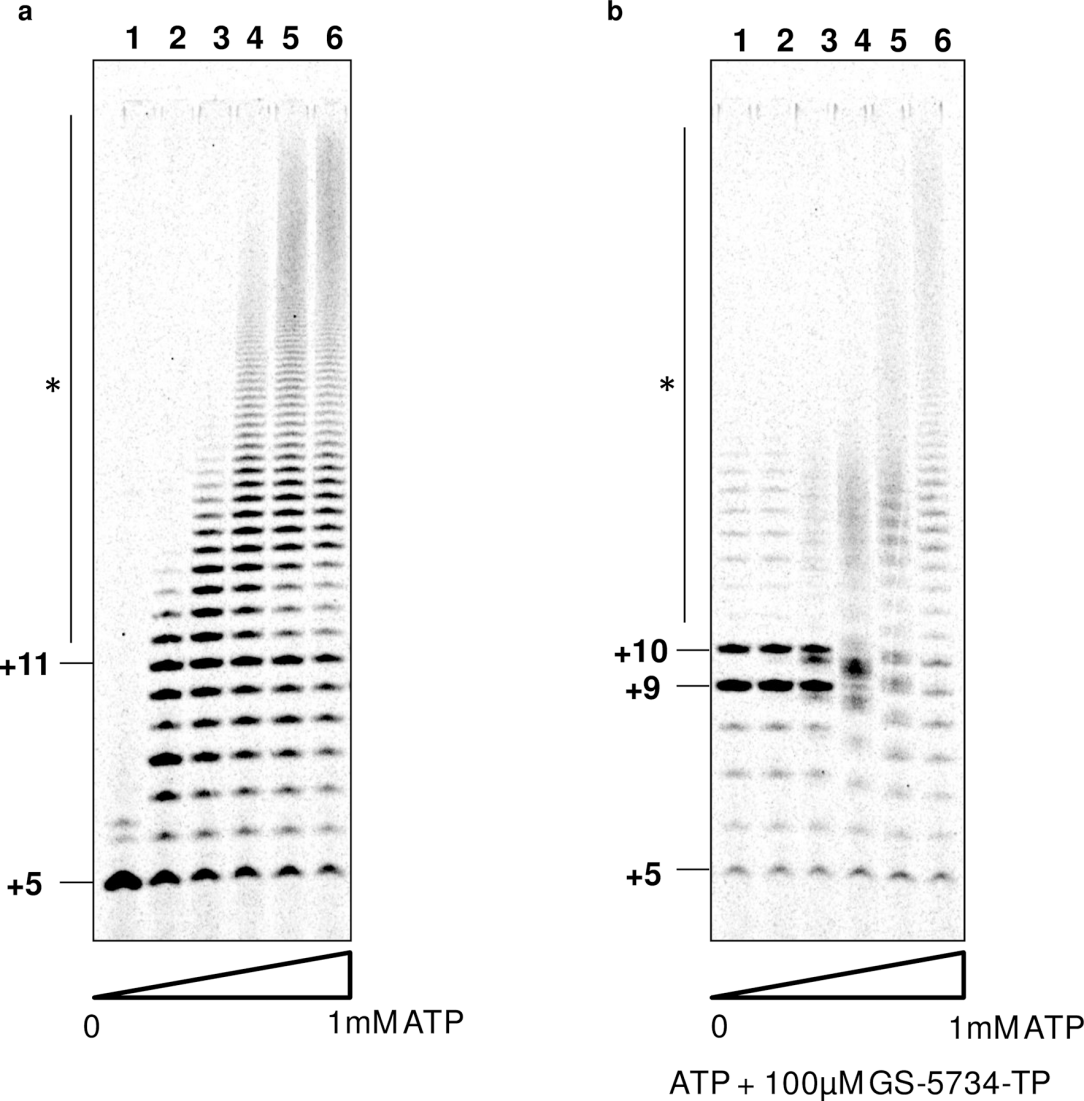
Factors driving and inhibiting polyadenylation of NiV polymerase. (a) NiV L(wt)-P, poly(U)_6_ template, and 4-mer primer were incubated with α^33^P-GTP with variable concentrations of ATP from 100 nM to 1 mM (lanes 1–6: 0, 0.1, 1, 10, 100, 1000 μM ATP). (b) Same as (a), except 100 μM GS-5734-TP as added to each reaction condition.

The minimum number of uridines within a template sequence required to trigger polyadenylation is not known for NiV polymerase. Our results showed that, within the natural M-F GE sequence six uridines signaled polyadenylation and that enzyme stuttering was more pronounced in the absence of the AAUG sequence. Based on this observation, we varied the number of uridines on the poly(U) tract template to understand the minimum number of uridines required for polyadenylation, while keeping the rest of the sequence unchanged (Fig 5A). For all templates, the 4-mer primer was converted into a 5-mer product with the addition of α^33^P-GTP (Fig 5B, lanes 1–6). Upon the addition of α^33^P-GTP + ATP, only templates containing between four and six uridines triggered polyadenylation (Fig 5B, lanes 7–9). These results indicate at least four consecutive uridines are required for polyadenylation. Templates containing one, two, or three uridines yielded predominantly products of expected size, 6, 7, and 8 nt, respectively (Fig 5B, lanes 10–12). All six templates contained a GA sequence directly downstream of the poly(U) tract. To determine the contribution to enzymatic stuttering of the next correct nucleotides, the same experiments were repeated with CTP + UTP (opposite GA on template) in addition to α^33^P-GTP + ATP. The main effect of adding the next correct nucleotides was a marked reduction in polyadenylation for the poly(U)_4_ and poly(U)_5_ templates (Fig 5C). In addition to the upstream and downstream sequences, we also found that ATP concentration was critical for polyadenylation. At 100 nM ATP, the efficiency of polyadenylation with the pol(U)_6_ template was minimal (Fig 6A, lane 1). The efficiency of polyadenylation and the size of the products gradually increased with increasing concentrations of ATP up to 1 mM (Fig 6A, lanes 2–6). With requirements for such high ATP concentrations for enzymatic stuttering, we hypothesized that ATP analogs acting as chain terminators might block polyadenylation by competing with natural ATP. This was confirmed by adding GS-5734-TP to the reaction at a physiologically relevant concentration of 100 μM, which resulted in significant reduction in polyadenylation even in the presence of 1 mM ATP (Fig 6B). This result suggests that chain-terminating adenosine analogs might also inhibit transcription termination by blocking polymerase stuttering.

## Discussion

This study is the first report of the expression, purification, and biophysical and biochemical characterization of an active recombinant paramyxovirus polymerase. Using an insect cell expression system with an affinity tag on the L protein, we purified the NiV L(wt)-P, NiV L(H1347A)-P, and NiV L(D832A-N833A)-P by FLAG purification followed by SEC. The SEC profile revealed a heterogenous preparation with a significant peak near the void volume for all three L-P complexes (S1 Fig). Despite employing multiple chromatographic techniques, we were unable to separate this aggregated population from a more monodisperse population. We verified the sequence identity of the L and P proteins by mass spectrometry and subjected the purified protein to analysis by SDS-PAGE, which revealed bands of nearly equal intensity for L and P proteins (Figs 1C and S2). Preparations of NiV L(wt)-P, NiV L(H1347A)-P and NiV L(D832A-N833A)-P showed minor bands likely attributed to HSP90 and HSC70 proteins (S2 Fig). Previous work has shown that HSP90 may work as a chaperone [27] for the polymerase complex; future studies must critically evaluate the role of chaperone proteins with recombinant NiV L-P.

Bioinformatic, biochemical, and biophysical analyses indicate that paramyxoviral P proteins contain disordered and folded regions [28,29]. It has also been reported that the multimerization domain of NiV P protein alone adopts a long, parallel, tetrameric, coiled coil structure with an additional alpha-helical cap while others have reported it forms an elongated coiled-coil trimer [20,22]. Structural studies on the P protein fragment were completed on P protein expressed in *Escherichia coli* cells without L or N proteins, which contrasts with the work presented here [20,22]. It has been shown that fragments of NNS RNA virus P proteins oligomerize with a range of stoichiometries [20,28,30–32]. When complexed with NiV L protein, the NiV P protein may adopt a different oligomeric state than when in solution alone. An understanding of the L-P interaction for NiV and other related viruses will not only provide insights into viral replication but will also inform the design of therapeutic compounds to interfere with replication by disrupting this interaction.

To further probe molecular arrangement and monodispersity of the NiV L-P complex, we visualized the purified NiV L-P complex using negative-stain EM followed by 2D class averaging. Our data revealed two main populations of globular particles, ranging in size from 5–11 nm; four representative class averages are displayed (Fig 1D). An additional subset of aggregated particles not included in class averages, was also observed (S1 Fig). The globular particles adopted a core ring-like structure with some class averages showing an interior density (Fig 1D). These structures resemble those of the VSV L RdRp when visualized by negative-stain EM [17,18]. We also clearly saw particles smaller than expected for an L-P complex of this molecular weight. Our data are not of high enough resolution to provide insights into the secondary structure of the P protein or the role, if any, of the NiV L-P quaternary structure, which remains an important question for NiV and other paramyxoviral polymerases. Our negative-stain EM data do not clearly show the P protein, which may be due to its overall intrinsic disorder, size, and the low resolution of our micrographs. This absence of any defined P protein structure may account for the smaller particle sizes. Overall, our interpretation is the NiV L-P complex exhibits a range of molecular arrangements with similarities to related polymerases.

Given the molecular architectures we observed by negative-stain EM, we wanted to use recombinant NiV L-P complex to understand the molecular interactions between the polymerase and its RNA substrate. More specifically, we present here how NiV L-P recognizes the 3′-end of the viral genome, and how the polymerase transitions from initiation of RNA synthesis to elongation mode. The *de novo* RNA synthesis assay showed that high concentrations of CTP and ATP are needed for the initiation step (Fig 2C), which is consistent with the role of these two nucleotides in initial primer bond formation given the *Le* promoter sequence at the 3′-end of the template (Fig 2A). The 1,000-fold reduction in *K*_*m*_ for ATP in the primer extension assay confirmed that the enzyme had transitioned to elongation mode, with faster and more efficient RNA synthesis (S8 Fig). The change in enzyme kinetics associated with transition from initiation to elongation is reminiscent of other viral RNA polymerases [33]. The formation of 10- and 11-mer RNA products by NiV L-P in the *de novo* RdRp assay using a 12-mer template was not completely unexpected since a +3-initiation site had previously been reported for the related RSV polymerase [23]. Since the majority products in the primer extension assay were also 10- and 11-mer (Fig 3), we concluded that these shorter RNAs were not generated by +3-initiation, but instead mainly by RNA synthesis starting at the +1-position and premature dissociation at the 5′-end of the template. The lack of full length 12-mer product is most likely due to the enzyme dissociating from the 5′-end of the template. The adenine scanning experiment showed that, except for position +5, all other first six bases or positions on the template are important for efficient *de novo* initiation of RNA synthesis. The observation that NiV L-P can also accommodate an RSV template indicates that, rather than recognizing a specific sequence involving each nucleobase, the polymerase complex may be sensitive to a certain sequence environment that remains to be further explored. Likewise, it has recently been shown that recombinant rabies virus polymerase also recognizes the VSV promoter sequence [34]. As a follow-up to this work, measuring the direct binding interaction between NiV polymerase and various RNA promoter sequences would help to better define the specificity of promoter recognition.

Interestingly, the H1347A mutation in the HR motif of the capping domain conferred a 2-fold loss in RdRp activity (Fig 2A), and this effect was almost completely reverted in the primer extension assay (Fig 3B). This finding suggests some level of cooperativity between RdRp and the capping domain during the early stages of RNA synthesis, which could be a shared function with other NNS RNA viruses [35]. The high degree of sequence conservation in the CRV capping motif among NNS RNA virus L proteins suggests that NiV L-P should function as a capping enzyme (Fig 1B). Since PRNTase activity in L proteins from viruses other than rhabdoviruses has never been established [36–38], an extension to the work present here would be to study the molecular requirements in NiV L-P for mRNA capping.

Another aim of our experiments was to understand which sequence elements in NiV gene junctions control the balance between replication and transcription, given that both events are initiated by the same genomic RNA template sequence but lead to two different RNA products: full-length antigenomic RNA and shorter mRNAs. It is already known from other NNS RNA viruses that intracellular levels of N protein favor polymerase replication through nucleocapsid assembly, but do not directly inhibit transcription [39,40]. Another important mechanism regulating the efficiency of transcription of NNS RNA virus genomes is polyadenylation by polymerase stuttering.

In NiV, GE sequences located within gene junctions contain five or six consecutive uridine repeats [10]. We found that NiV L-P recognizes the M-F GE sequence containing six uridines to produce polyadenylated RNA, and that the efficiency of stuttering is significantly increased when the sequence upstream of the poly(U)_6_ tract is removed (Fig 4). Four consecutive uridines were sufficient to trigger polymerase slippage along the template when ATP was provided at a high concentration as the first correct nucleotide in the enzymatic reaction (Fig 5). Adding the next correct nucleotides increased the amount of read-through products relative to poly(A) RNAs. This observation is important to understand how NiV and probably other NNS RNA viruses have evolved by optimizing polymerase slippage along template strands containing uridine-rich regions to regulate transcriptional termination and prevent aberrant polyadenylation. VSV requires a poly(U)_7_ tract for polyadenylation, whereas RSV polymerase only needs a four-uridine repeat in its *cis*-acting GE region [41]. In VSV, the spacing between the GS and GE sequences affects mRNA synthesis by modulating transcription termination [42]. Although we cannot exclude that the GS signal also regulates transcription termination in NiV, it is tempting to hypothesize that polymerases from different NNS RNA virus families respond differently to intergenic sequences. Even within the same polymerase, the response to intergenic sequences is different if transcribing or replicating. Uridine-rich regions are not unique to GE signals. The 3′-terminal leader and trailer promoter regions in pneumovirus genomes also contain a poly(U) tract, which has been shown to trigger enzyme stuttering [43]. However, we found that NIV L-P is much more prone to polyadenylation than RSV L-P (Fig 4B). Furthermore, RSV L-P reads through its leader promoter RNA without significant polyadenylation (S10 Fig). We think these results explain why pneumoviruses contain a polyU-tract sequence in their genomic and antigenomic promoters, which are not found in paramyxoviruses, further suggesting different mechanisms for controlling polyadenylation between proteins from these two virus families. Since poly(U) tracts are also found upstream of gene-start sequences and in editing sites [7,44–48], it is likely that the data presented here could also help to study how polymerase stuttering in coding and non-coding uridine-rich regions affect transcription initiation and mRNA editing. Ultimately, more work will be needed to further elucidate the role of polyadenylation signals in the regulation of NNS RNA virus polymerase activities.

Nucleoside analogs are the backbone of most antiviral therapies. Recently, two nucleoside analogs have been reported as potent paramyxovirus inhibitors. R1479, a 4′azido-cytidine analog previously developed for the treatment of hepatitis C virus infection, also inhibits NiV replication with an *in vitro* potency of 1–5 μM [24]. The anti-Ebola molecule GS-5734 is a broad-spectrum clinical-stage adenosine analog that also targets NiV, with an *in vitro* antiviral potency of 0.05 μM [25,26]. In our study, we showed that the respective TP forms of both nucleoside analogs are efficient substrates incorporated into viral RNA by NiV L-P (Fig 3). In our assay conditions, a single incorporation of R1479-TP was not sufficient to block RNA synthesis, which indicates that this cytidine analog is not an immediate chain terminator. This contrasts with the immediate chain termination effect of R1479-TP against hepatitis C virus polymerase [49]. In the case of GS-5734-TP, three incorporation events were required to prevent full-length RNA synthesis (Fig 3). This delayed or leaky chain termination effect on NiV L-P has also been described for RSV polymerase [26]. In addition, we observed that GS-5734-TP also inhibits transcription termination by competing with natural ATP and preventing polyadenylation (Fig 6). This additional mechanism of action might explain why GS-5734 is more potent than R1479 in infected cells, and sheds light on the potential use of adenosine analogs as dual replication and transcription termination inhibitors. Studies such as this one not only provide valuable tools to evaluate the mechanism of action of existing clinically relevant RNA polymerase inhibitors, but could also be used to develop novel biochemical assays to discover inhibitors of NiV and related paramyxoviruses of public health importance.

## Materials and methods

### Chemicals

R1479-TP and GS-5734-TP were synthesized at Alios BioPharma (South San Francisco, CA). Oligonucleotides were synthesized at Dharmacon, Inc (Lafayette, CO). Their sequences with analysis of secondary structures can be found in S11 Fig. The sequence of the oligonucleotides used as product size markers in sequencing urea PAGE is as follows: 5'-ACCA-3' (4-mer), 5'-ACCAG-3' (5-mer), 5'-ACCAGA-3' (6-mer), and 5'-ACCAGACAAGGG-3' (12-mer).

### Molecular modeling

A homology-based three-dimensional structure of the NiV L protein was generated using RaptorX [50]. To generate an accurate model, approximately 100 residues were removed from the sequence. The molecular graphics of the resultant structure (blue) were generated using the University of California at San Francisco Chimera package [51].

### Cloning and production of NiV L-P proteins

The codon-optimized ORFs for the NiV L and P proteins (Bangladesh genotype, Genbank accession AY988601.1) (Genscript, Piscataway, NJ) were a gift from Michael Lo, cloned separately into pFastBac Dual expression vector. The 3XFLAG tag was added to the N-terminus of the NiV L protein by nested polymerase chain reaction (PCR) (two rounds of PCR with overlapping oligos containing the 3XFLAG tag; Eton Bioscience, San Diego, CA) with PrimeSTAR DNA polymerase (Takara Bio, Shiga, Japan). The 5′ SacI / HindIII region from the untagged NiV L was then replaced by the 3XFLAG-L PCR fragment. The SacI / XhoI fragment containing NiV P-His was cloned into SacI / XhoI cut pFBD-3xFlag-NiV L to make pFBD-3xFlag NiV L-P-His. The L protein, under the control of the polyhedron promoter, was cloned with an N-terminal 3X FLAG tag. The P protein, under the control of the P10 promoter, was cloned with a C-terminal hexahistidine tag. E. coli DH10Bac was transformed with the pFastBac Dual vector to yield bacmid DNA. A high-titer baculovirus stock was generated after transfection of bacmid DNA into Sf9 cells using Cellfectin. Mutagenesis of the L gene to generate NiV L(H1347A) and NiV L(D832A-N833A) was completed by amplifying the whole template DNA with complementary pairs of mutagenic oligonucleotides (Eton Bioscience) using Kapa HiFi HotStart DNA polymerase (Kapa Biosystems, Wilmington, MA), followed by full-length insert sequencing confirmation.

Two liters of Sf9 insect cells were infected with the baculovirus stock at a multiplicity of infection of 1 and harvested 72 hours post-infection by centrifugation for 10 minutes at 1000 × g The cells were resuspended in lysis buffer (50 mM Tris pH 7.4, 150 mM NaCl, 10% glycerol, 0.1% octyl β-D-glucopyranoside, 0.1% n-dodecyl β-D-maltoside, EDTA-free protease inhibitor, 10 U/mL benzonase), lysed by microfluidization, and clarified through centrifugation for 30 minutes at 14,000 × g. The clarified lysate was incubated with EZview Red ANTI-FLAG M2 Affinity gel, washed twice with lysis buffer, and the NiV polymerase complex was eluted using 3X FLAG peptide, and concentrated using Amicon Ultra Centrifugal Filters. The sample was further purified by size-exclusion chromatography using a Superdex 200 Increase 10/300 GL at a flow rate of 0.3 mL/min with a running buffer of 50 mM Tris pH 7.4, 150 mM NaCl, 10% glycerol, 0.1% octyl β-D-glucopyranoside, 0.1% n-dodecyl β-D-maltoside, 1 mM DTT. The protein was then concentrated using an Amicon Ultra Centrifugal Filter, subjected to SDS-PAGE, and quantified using a Bradford Assay. The bands associated with the L, P, and HSC70 proteins were digested with trypsin, chymotrypsin, and elastase followed by analysis by nano LC-MS/MS with a Waters NanoAcquity HPLC system interfaced to a ThermoFisher Q Exactive. The peptides were then loaded on a trapping column and eluted over a 75-μm analytical column at 350 nL/min. Both columns used were packed with Luna C18 resin (Phenomenex, Torrance, CA). The mass spectrometer was operated in data-dependent mode, with MS and MS/MS performed in the Orbitrap at 70,000 full-width at half-maximum (FWHM) resolution and 17,500 FWHM resolution, respectively. The fifteen most abundant ions were selected for MS/MS.

### Transmission electron microscopy and image analysis

Samples were prepared on continuous carbon films supported by nitrocellulose-coated 400-mesh copper grids (Ted Pella). A 3 μl drop of NiV L(wt)-P, diluted 60-fold in buffer (20 mM Tris pH 7.5, 10 mM KCl, 2 mM DTT, 6 mM MgCl_2_), was applied to a freshly plasma-cleaned grid, blotted to a thin film with filter paper and immediately stained with 1% (wt/v) uranyl formate. Electron microscopy was performed using an FEI Tecnai T12 electron microscope operating at 120keV equipped with an FEI Eagle 4k x 4k CCD camera. Images were collected at nominal magnifications of 110,000× (0.10 nm/pixel) and 67,000× (0.16 nm/pixel) using the automated image acquisition software package Leginon [52]. The images were acquired at a nominal underfocus of -0.9 μm to -1.8 μm and electron doses of ~25 e^-^/Å^2^.

Image processing was performed using the Appion software package[53]. Contrast transfer functions of the images were corrected using ctffind4 [54]. Individual particles in the 67,000× images were selected using automated picking protocols followed by several rounds of reference-free alignment and classification based on the XMIPP processing package to sort them into self-similar groups [55].

### Minigenome assay

BSRT7/5 cells were a gift from K. Conzelmann [56], and were propagated in DMEM supplemented with 5% FBS and 1 mg/mL G418 antibiotic. A Nanoluciferase-based NiV minigenome assay was adapted from a previously developed NiV minigenome assay [9]. Briefly, a bacteriophage T7 polymerase-based NiV minigenome was synthesized (Genscript, Piscataway, NJ) expressing a reporter fusion construct of Nanoluciferase (Promega, Madison, WI) and mNeonGreen fluorescent protein [57]. The open-reading frame encoding the reporter fusion protein was flanked by a T7 promoter, hammerhead ribozyme, NiV leader and N gene untranslated region at the 3′-end, with the NiV L gene untranslated region and genomic trailer, and a hepatitis delta virus ribozyme at the 5′-end. BSRT7/5 cells (1×10^4^) were seeded in 96-well plates overnight. The next day, NiV support plasmids consisting of 50, 32, and 50 ng/well of N, P, and L plasmids, respectively, and NiV minigenome (120 ng) prepared in RNase-free TE buffer, were mixed with 0.6 μL/well LT-1 transfection reagent (Mirus Bio, Madison, WI) and 10 μL Opti-MEM/well. Complexes were mixed and incubated for 30 minutes at room temperature before being added to cells. For negative controls, the L plasmid was substituted with an equivalent amount of pcDNA 3.1 plasmid expressing the red fluorescent protein mCherry (Clontech). At 48 hours post-transfection of minigenome and support plasmids, 50 μL of Nanoluciferase assay buffer solution (Promega) was added directly to each well. Well contents were transferred to 96-well opaque white plates, and after 3 minutes, luminescence was read on a plate reader using 0.1 msec integration time (HT-Synergy, Biotek, Winooski, VT).

### NiV and RSV RdRp assay

Unless otherwise specified, all NiV and RSV polymerase reactions consisted of 0.2 μM oligonucleotide template derived the NiV leader promoter, 0.2 μM recombinant L-P complex, with a buffer containing 20 mM Tris pH 7.5, 10 mM KCl, 2 mM DTT, 0.5% triton, 10% DMSO, 6mM MgCl_2_. Recombinant RSV L-P was produced through the co-expression of RSV L and P proteins in a baculovirus expression system, according to previously described procedures [23]. In the primer-dependent reaction, this was then combined with 200 μM primer. Reactions were initiated through addition of specific nucleoside TP for the template sequence to final volume of 10 μL and incubated at 30°C for 30 minutes. The radioisotope tracer in these reactions was α^33^P-GTP. Reactions were stopped with the addition of an equal volume of gel loading buffer (Ambion, Austin, TX) denatured at 95°C for 5 minutes, and run on a 22.5% polyacrylamide urea sequencing gel for 2 hours at 80W. The migration products were exposed to a phosphor-screen, scanned on Typhoon phosphorimager (GE Healthcare, Chicago, IL) and quantified using ImageQuant (GE Healthcare).

## Supporting information

S1 Fig Characterization of NiV polymerase by negative stain EM and purification by SEC. (a) Representative image at high magnification (110,000x) used to generate 2D classes (scale bar: 10 nm) (b) Representative SEC chromatogram of NiV L(wt)-P purification. The sample was run on Superdex 200 Increase 10/300 GL. Fractions associated with the peak at approximately 8mL were collected, pooled, and concentrated.
(TIF)

S2 Fig Analysis of purified NiV polymerase. SDS-PAGE analysis of NiV L(wt)-P, NiV L(H1347A)-P, and NiV L(D832A-N833A)-P.
(TIF)

S3 Fig Characterization of purified NiV polymerase by LC-MS/MS. (a) Coverage map of NiV L peptides identified after proteolytic digestion and ionization (b) Coverage map of NiV P peptides identified after proteolytic digestion and ionization (c) Coverage map of heat shock cognate 70 protein peptides identified after proteolytic digestion and ionization (d) Example product ion spectrum, for NiV L(wt)-P and (e), an additional, example product ion spectrum for NiV L(wt)-P.
(TIF)

S4 Fig *De novo* RNA synthesis by NiV L-P and requirements for ATP and CTP to support RNA synthesis. (a) Product sizes are indicated using a 4-5-6-mer combined RNA size marker (lane M). NiV L(wt)-P and template were incubated in the presence of α^33^P-GTP tracer, a fixed ATP concentration, and CTP concentrations ranging from 0 to 1000μM (lanes 1–6). NiV L(wt)-P and template were incubated in the presence of α^33^P-GTP tracer, a fixed CTP concentration (1000 μM), and ATP concentrations ranging from 0 to 1000μM (lanes 7–12). (b) The concentrations of ATP and CTP loaded on the gel represented in (a).
(TIF)

S5 Fig Comparing template requirements for *de novo* RNA synthesis by NiV L-P. (a) The 12-mer RNA template from the leader promoter region of the NiV genome and an 11-mer RSV-template derived from the leader promoter region of the RSV genome. (b) Product sizes are indicated using a 4-5-6-mer combined RNA size marker (lane M). NiV L(wt)-P and either NiV or RSV template were incubated in the presence of α^33^P-GTP tracer (lanes 1 and 3), or with α^33^P-GTP + ATP + CTP (lanes 2 and 4). The total RdRp activity (%) for both templates was quantified and expressed as a bar graph.
(TIF)

S6 Fig Analysis of RNA synthesis products through introduction of adenine at the first nine bases of the promoter sequence (used to generate data in Fig 2D). (a) Product sizes are indicated using a 4-5-6-mer combined RNA size marker (lane M). NiV L(wt)-P and template described in (b) were combined with α^33^P-GTP, ATP, and CTP. (b) Templates loaded in specific wells in gel image presented in (a).
(TIF)

S7 Fig *De novo* and primer-dependent RNA synthesis by NiV L-P. (a) The 12-mer RNA template from the leader promoter region. (b) Product sizes are indicated using a kinase-labeled set of four combined oligonucleotides: 4-, 5-, 6-, and 12-mer primer (lane M). NiV L(wt)-P and template were incubated in the presence of α^33^P-GTP tracer (lane 1), α^33^P-GTP + ATP + CTP (lane 2), α^33^P-GTP + primer (lane 3), or α^33^P-GTP + ATP + CTP + primer (lane 4). NiV L(D832A-N833A)-P mutant and template were incubated in the presence of α^33^P-GTP tracer (lane 5), α^33^P-GTP + ATP + CTP (lane 5), α^33^P-GTP + primer (lane 7), or α^33^P-GTP + ATP + CTP + primer (lane 8). NiV L(H1347A)-P and template were incubated in the presence of α^33^P-GTP tracer (lane 9), α^33^P-GTP + ATP + CTP (lane 10), α^33^P-GTP + primer (lane 11), or α^33^P-GTP + ATP + CTP + primer (lane 12). (c) Table showing enzyme, NTPs, and primers along with well number.
(TIF)

S8 Fig Primer-dependent RNA synthesis by NiV L-P. (a) A 12-mer template with a short 4-nucleotide prime was used to analyze the *K*_*m*_ requirement for ATP. (b) A kinase-labeled primer was used as a marker to estimate size of extension products (lane M). NiV L(wt)-P, template, and primer were incubated in the presence of α^33^P-GTP tracer (lane 1), and with α^33^P-GTP combined with variable concentrations of ATP from 1nM to 1μM (lanes 2–8: 0, 0.001, 0.004, 0.01, 0.04, 0.01, 0.3, and 1μM ATP). (c) Data from multiple experiments was combined to generate a plot of RdRp Activity (%) vs ATP (μM), followed by a calculation of the Michaelis constant (*K*_*m*_).
(TIF)

S9 Fig Primer-dependent RNA synthesis and inhibition by NiV L-P. (a) A 12-mer template with four bases complementary to a short 4-nucleotide primer was used to analyze primer-extension activity of NiV L(wt)-P. Blue and green arrows indicate incorporation sites for 3′dATP or 3′dCTP at positions +6 or +7, respectively. (b) NiV L(wt)-P, template, and primer were incubated in the presence of α^33^P-GTP tracer (lane 1), α^33^P-GTP + ATP + CTP (lane 2), α^33^P-GTP + ATP + R1479-TP (lane 3), α^33^P-GTP + ATP + 3′dCTP (lane 4), α^33^P-GTP + CTP + GS-5734-TP (lane 5), and α^33^P-GTP + 3′dATP (lane 6).
(TIF)

S10 Fig *De novo* and primer-dependent synthesis by RSV L-P using the RSV *Le* promoter sequence template containing a poly(U)_6_ tract. (a) RSV L-P, an 18-mer template, were incubated in the presence of α^33^P-GTP tracer (lane 1) or α^33^P-GTP + ATP + CTP (lane 2). (b) RSV L-P, an 18-mer template, and primer were incubated in the presence of α^33^P-GTP tracer (lane 1), α^33^P-GTP + ATP (lane 2), or α^33^P-GTP + ATP + UTP.
(TIF)

S11 Fig Analysis of secondary structure in RNA templates. (a) The RNA templates used in RNA synthesis reactions with NiV with free energies associated with secondary structure formation (kcal/mol). (b) Predicted secondary structures for RNA.
(TIF)
