# Supplementary figures and images for: Initiation, extension, and termination of RNA synthesis by a paramyxovirus polymerase
Source: PLoS Pathog. 2018 Feb 9;14(2):e1006889. doi: 10.1371/journal.ppat.1006889 (PMC5823471; doi:10.1371/journal.ppat.1006889)

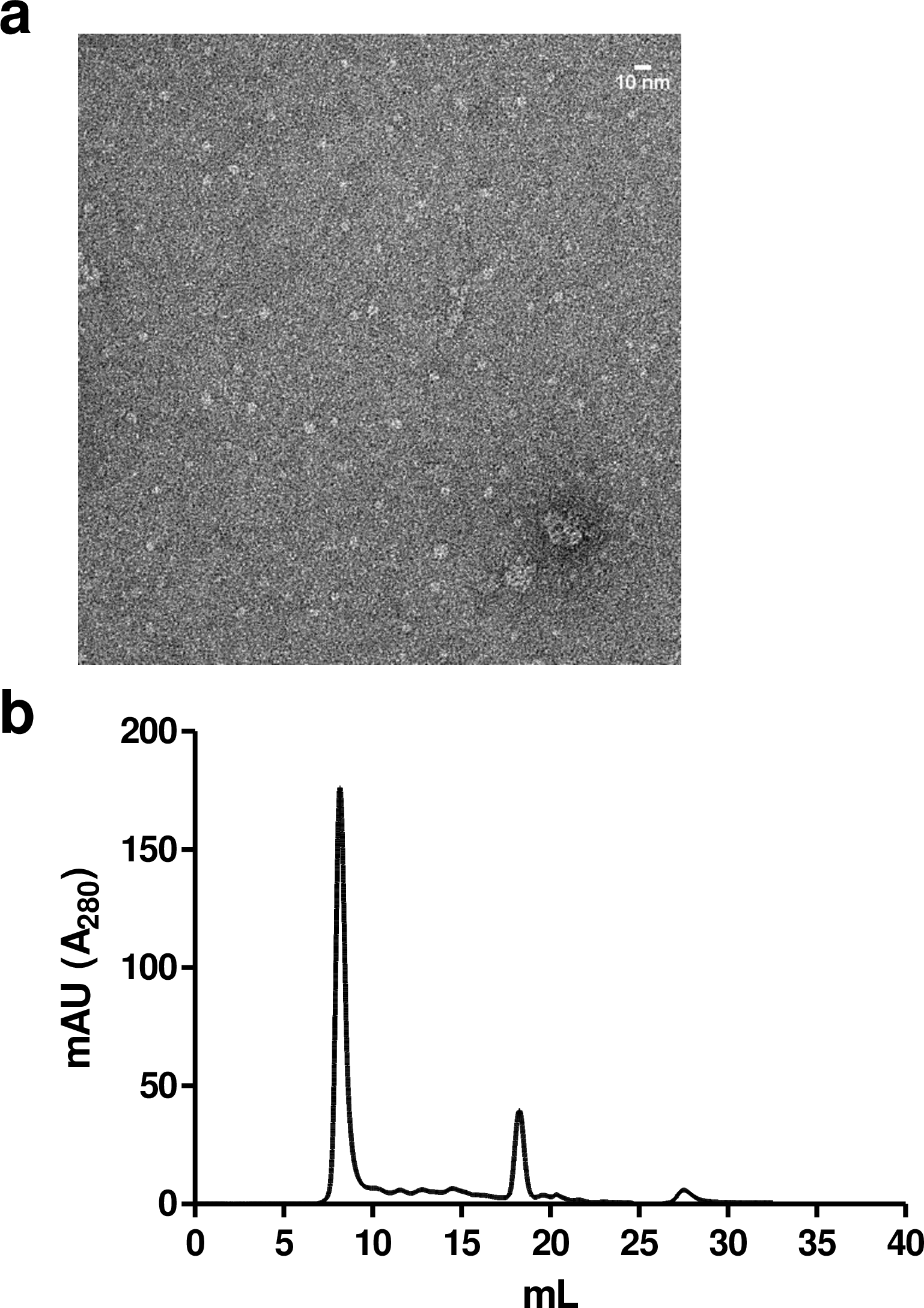

Supplement: S1 Fig — (a) Representative image at high magnification (110,000x) used to generate 2D classes (scale bar: 10 nm) (b) Representative SEC chromatogram of NiV L(wt)-P purification. The sample was run on Superdex 200 Increase 10/300 GL. Fractions associated with the peak at approximately 8mL were collected, pooled, and concentrated. (TIF) [file ppat.1006889.s001.tif]

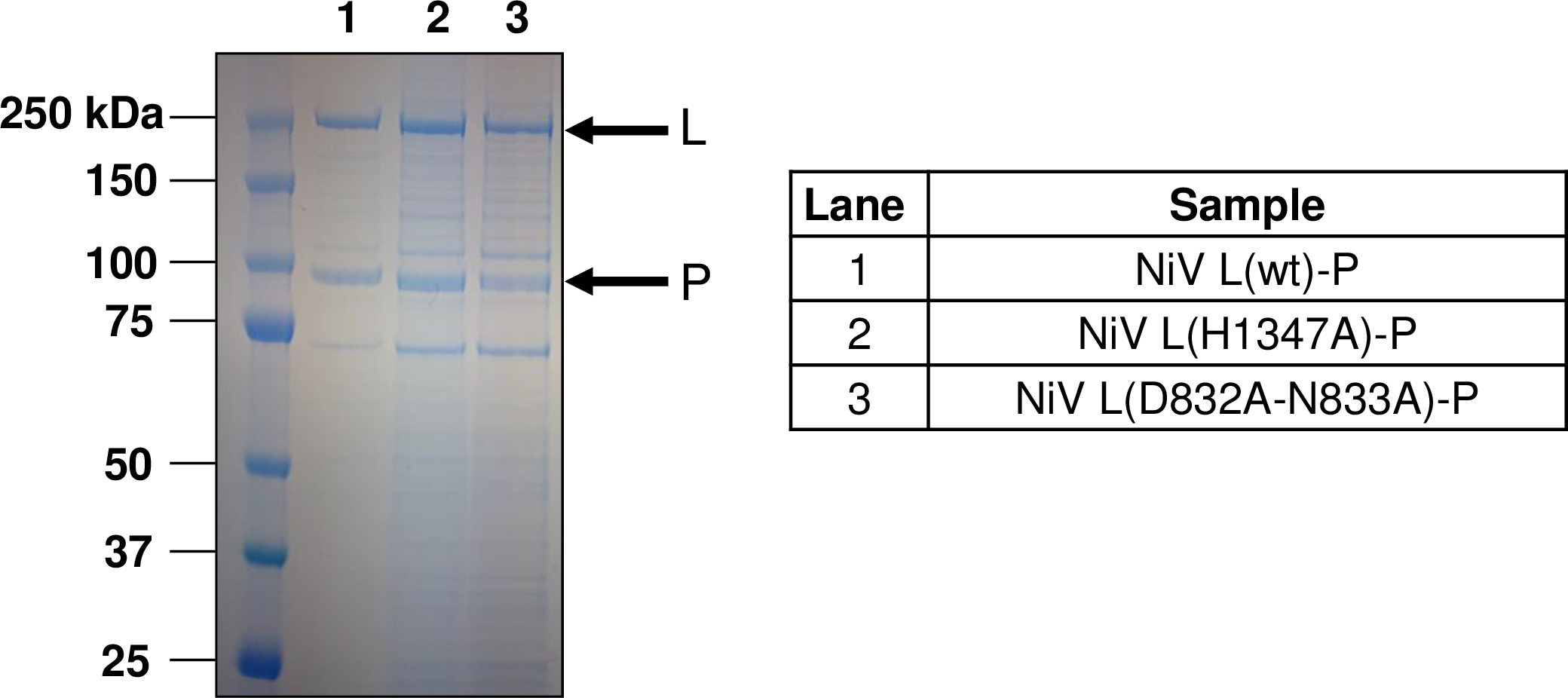

Supplement: S2 Fig — SDS-PAGE analysis of NiV L(wt)-P, NiV L(H1347A)-P, and NiV L(D832A-N833A)-P. (TIF) [file ppat.1006889.s002.tif]

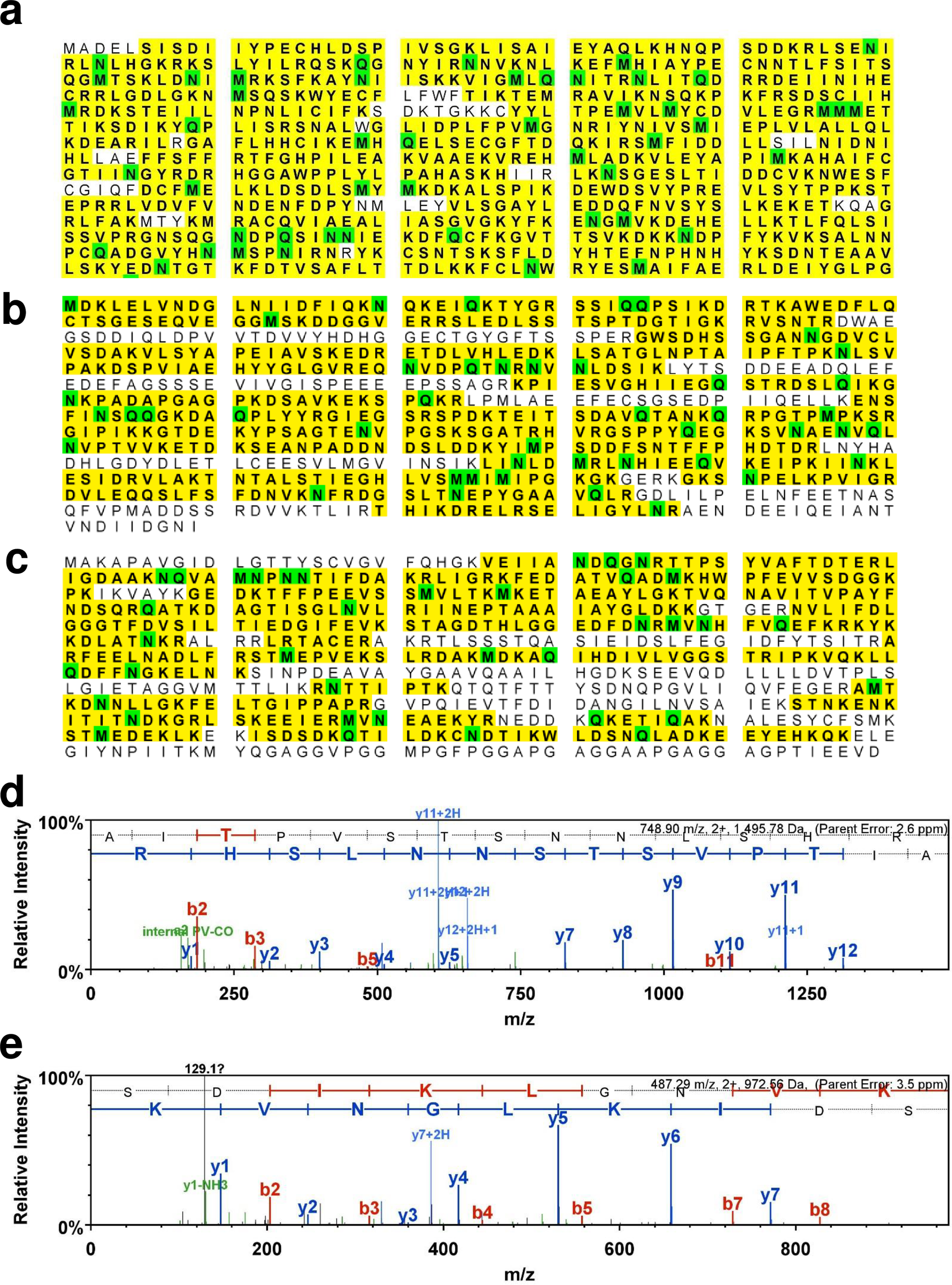

Supplement: S3 Fig — (a) Coverage map of NiV L peptides identified after proteolytic digestion and ionization (b) Coverage map of NiV P peptides identified after proteolytic digestion and ionization (c) Coverage map of heat shock cognate 70 protein peptides identified after proteolytic digestion and ionization (d) Example product ion spectrum, for NiV L(wt)-P and (e), an additional, example product ion spectrum for NiV L(wt)-P. (TIF) [file ppat.1006889.s003.tif]

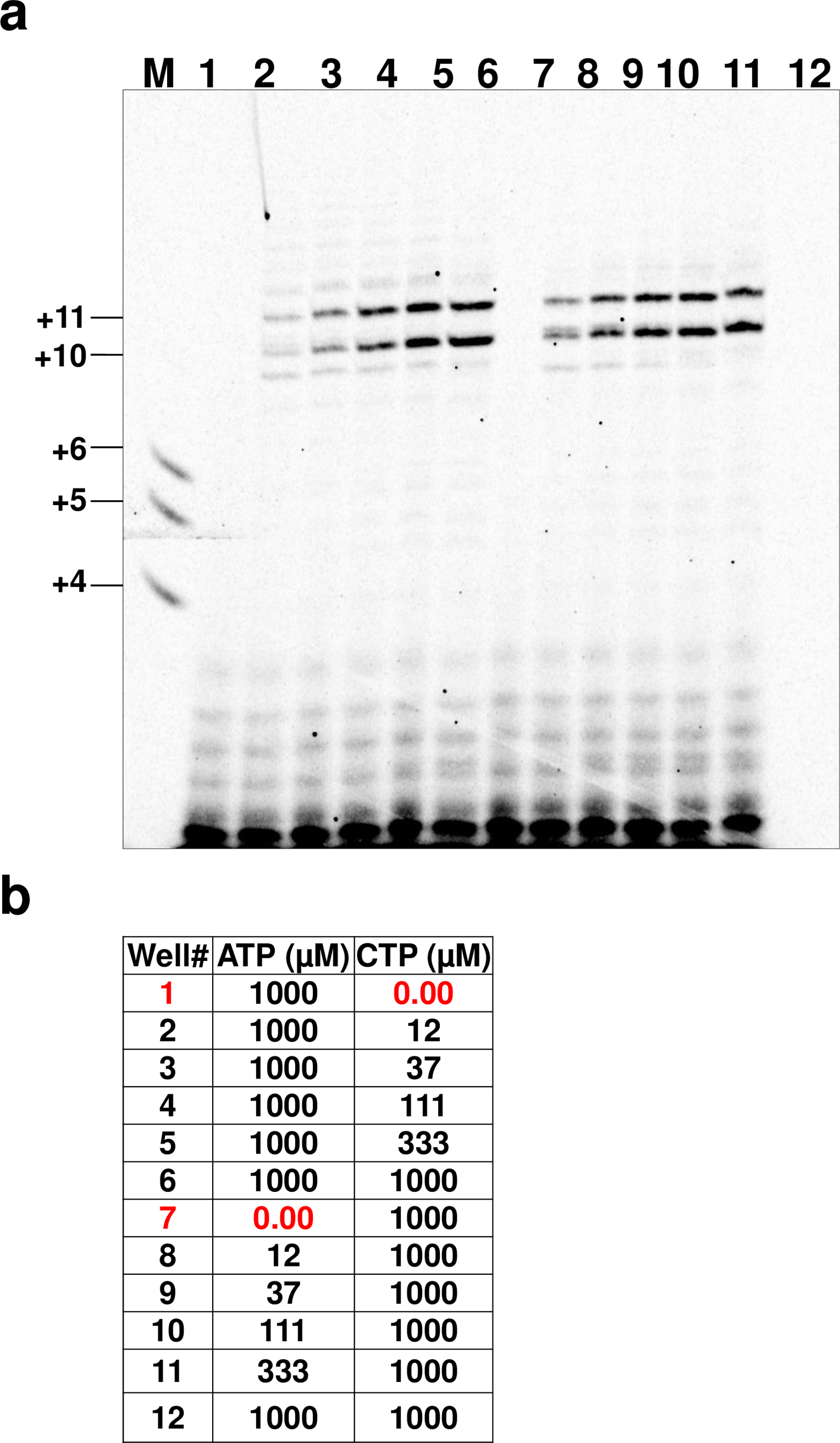

Supplement: S4 Fig — (a) Product sizes are indicated using a 4-5-6-mer combined RNA size marker (lane M). NiV L(wt)-P and template were incubated in the presence of α33P-GTP tracer, a fixed ATP concentration, and CTP concentrations ranging from 0 to 1000μM (lanes 1–6). NiV L(wt)-P and template were incubated in the presence of α33P-GTP tracer, a fixed CTP concentration (1000 μM), and ATP concentrations ranging from 0 to 1000μM (lanes 7–12). (b) The concentrations of ATP and CTP loaded on the gel represented in (a). (TIF) [file ppat.1006889.s004.tif]

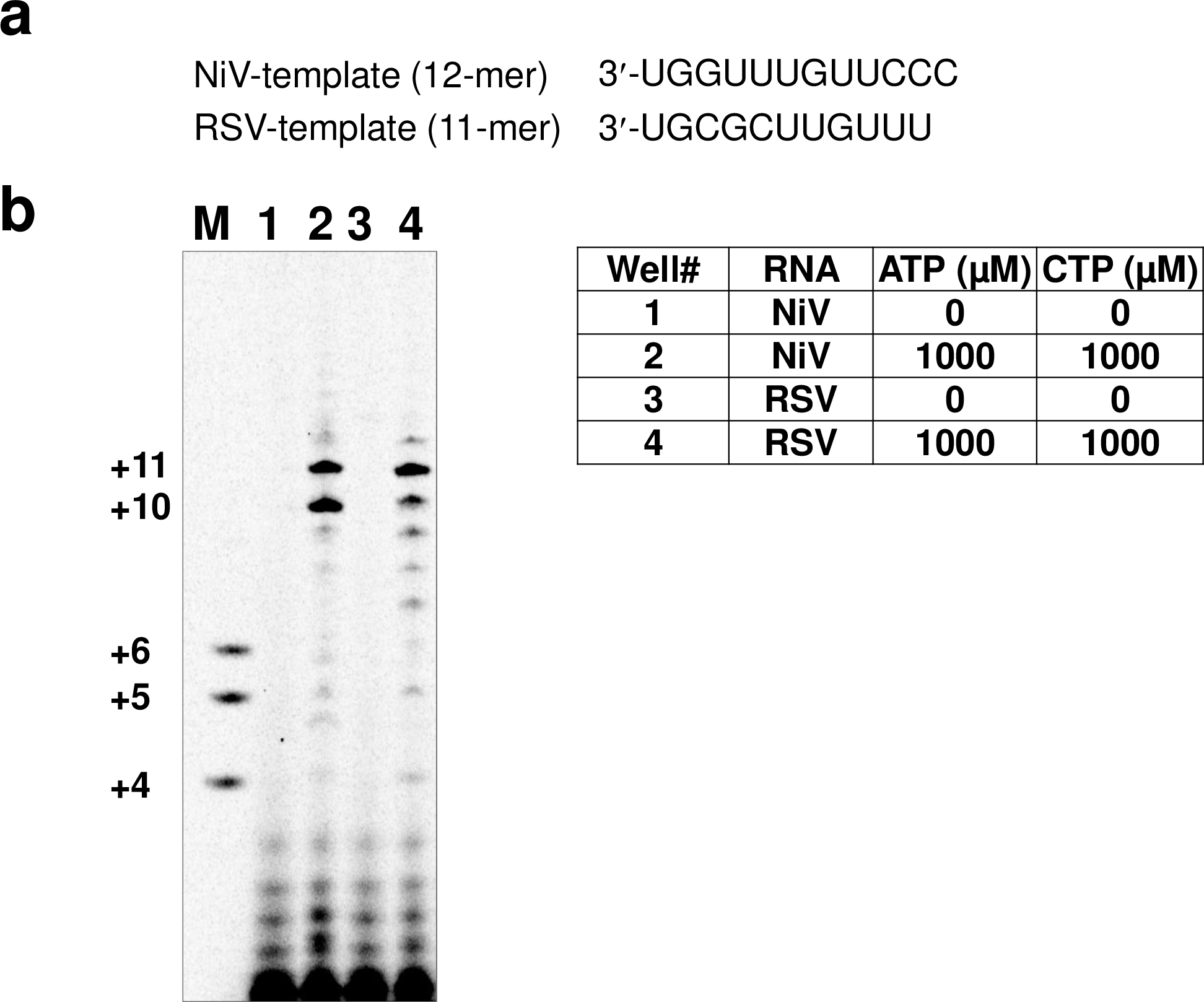

Supplement: S5 Fig — (a) The 12-mer RNA template from the leader promoter region of the NiV genome and an 11-mer RSV-template derived from the leader promoter region of the RSV genome. (b) Product sizes are indicated using a 4-5-6-mer combined RNA size marker (lane M). NiV L(wt)-P and either NiV or RSV template were incubated in the presence of α33P-GTP tracer (lanes 1 and 3), or with α33P-GTP + ATP + CTP (lanes 2 and 4). The total RdRp activity (%) for both templates was quantified and expressed as a bar graph. (TIF) [file ppat.1006889.s005.tif]

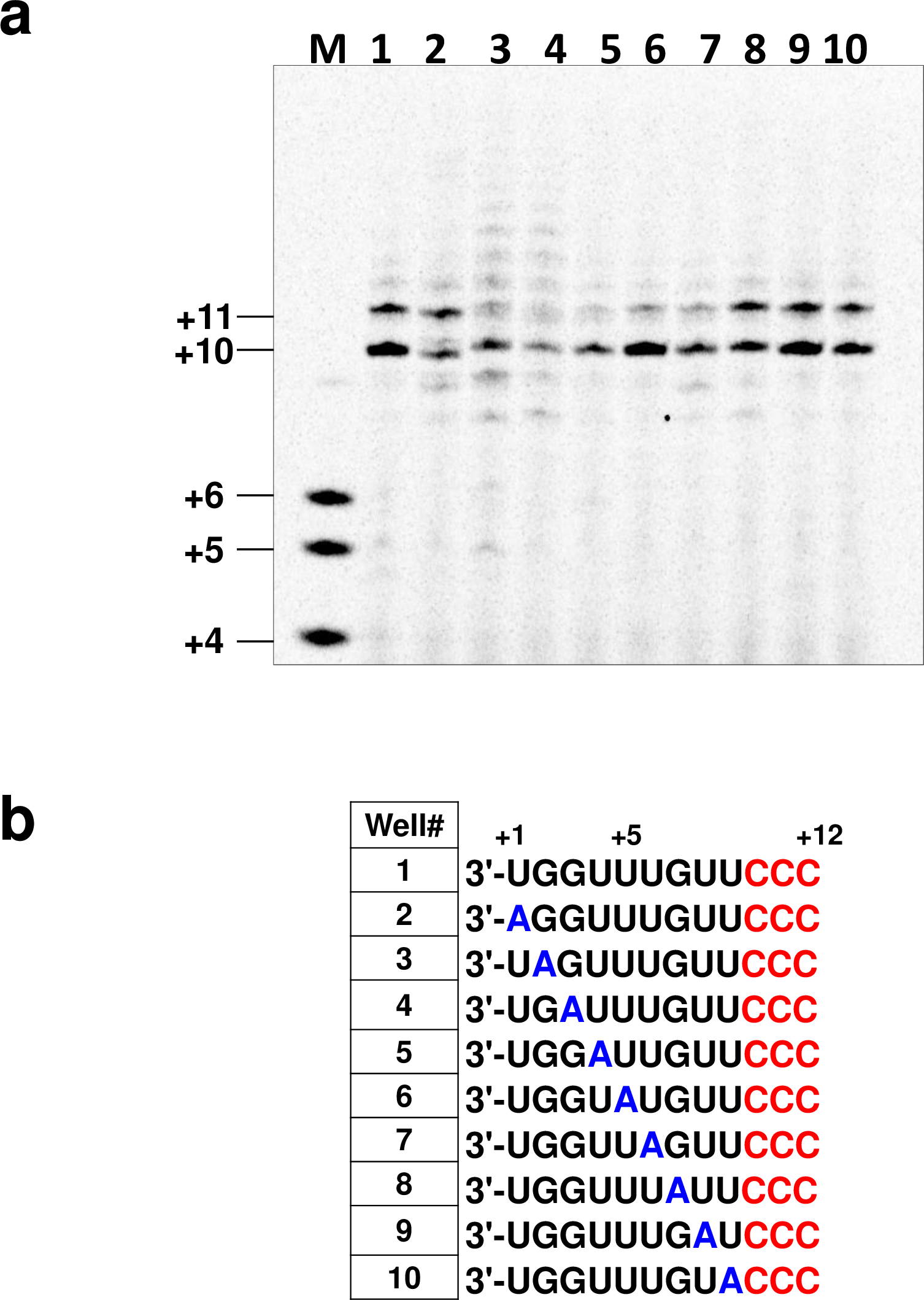

Supplement: S6 Fig — (a) Product sizes are indicated using a 4-5-6-mer combined RNA size marker (lane M). NiV L(wt)-P and template described in (b) were combined with α33P-GTP, ATP, and CTP. (b) Templates loaded in specific wells in gel image presented in (a). (TIF) [file ppat.1006889.s006.tif]

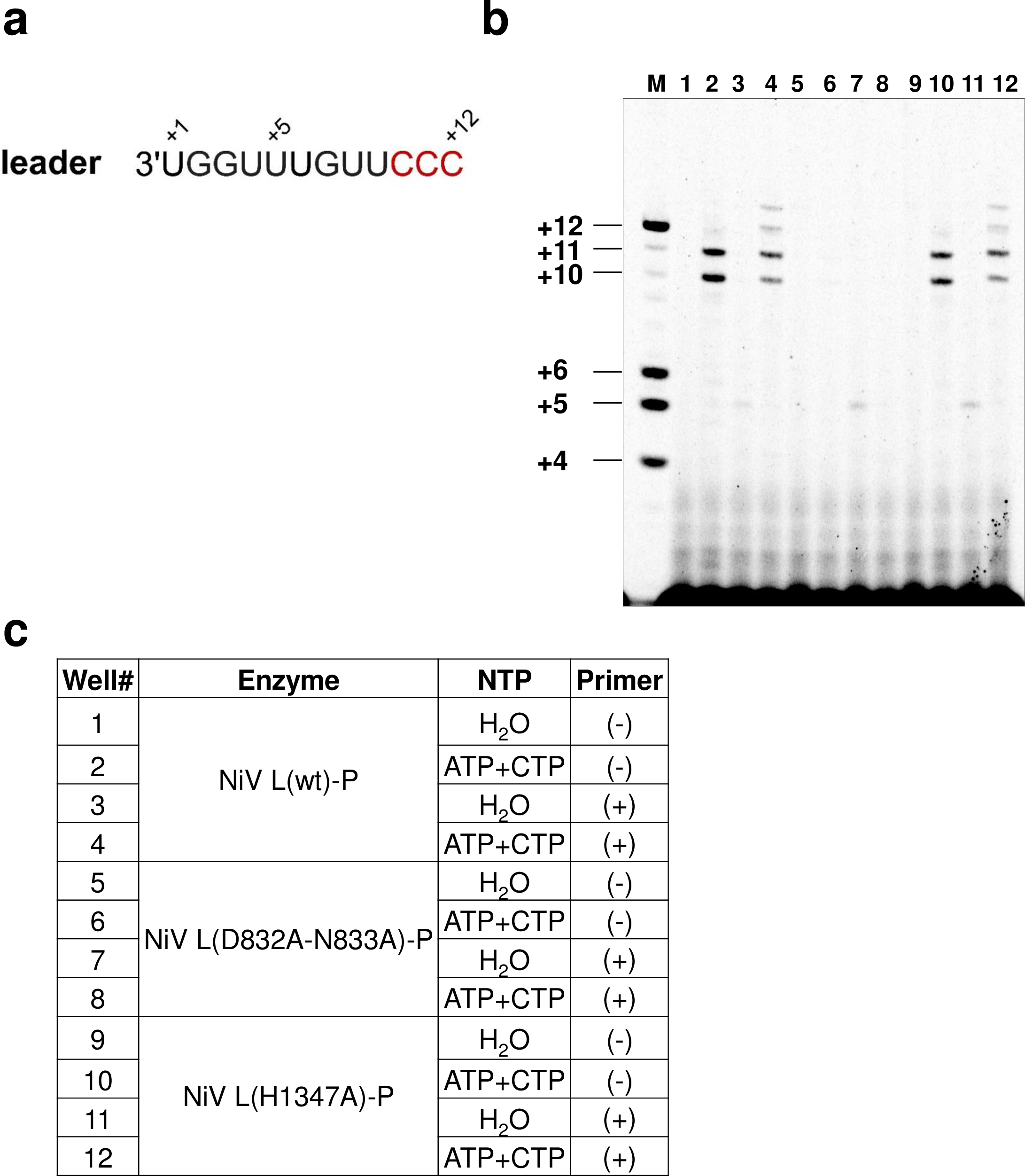

Supplement: S7 Fig — (a) The 12-mer RNA template from the leader promoter region. (b) Product sizes are indicated using a kinase-labeled set of four combined oligonucleotides: 4-, 5-, 6-, and 12-mer primer (lane M). NiV L(wt)-P and template were incubated in the presence of α33P-GTP tracer (lane 1), α33P-GTP + ATP + CTP (lane 2), α33P-GTP + primer (lane 3), or α33P-GTP + ATP + CTP + primer (lane 4). NiV L(D832A-N833A)-P mutant and template were incubated in the presence of α33P-GTP tracer (lane 5), α33P-GTP + ATP + CTP (lane 5), α33P-GTP + primer (lane 7), or α33P-GTP + ATP + CTP + primer (lane 8). NiV L(H1347A)-P and template were incubated in the presence of α33P-GTP tracer (lane 9), α33P-GTP + ATP + CTP (lane 10), α33P-GTP + primer (lane 11), or α33P-GTP + ATP + CTP + primer (lane 12). (c) Table showing enzyme, NTPs, and primers along with well number. (TIF) [file ppat.1006889.s007.tif]

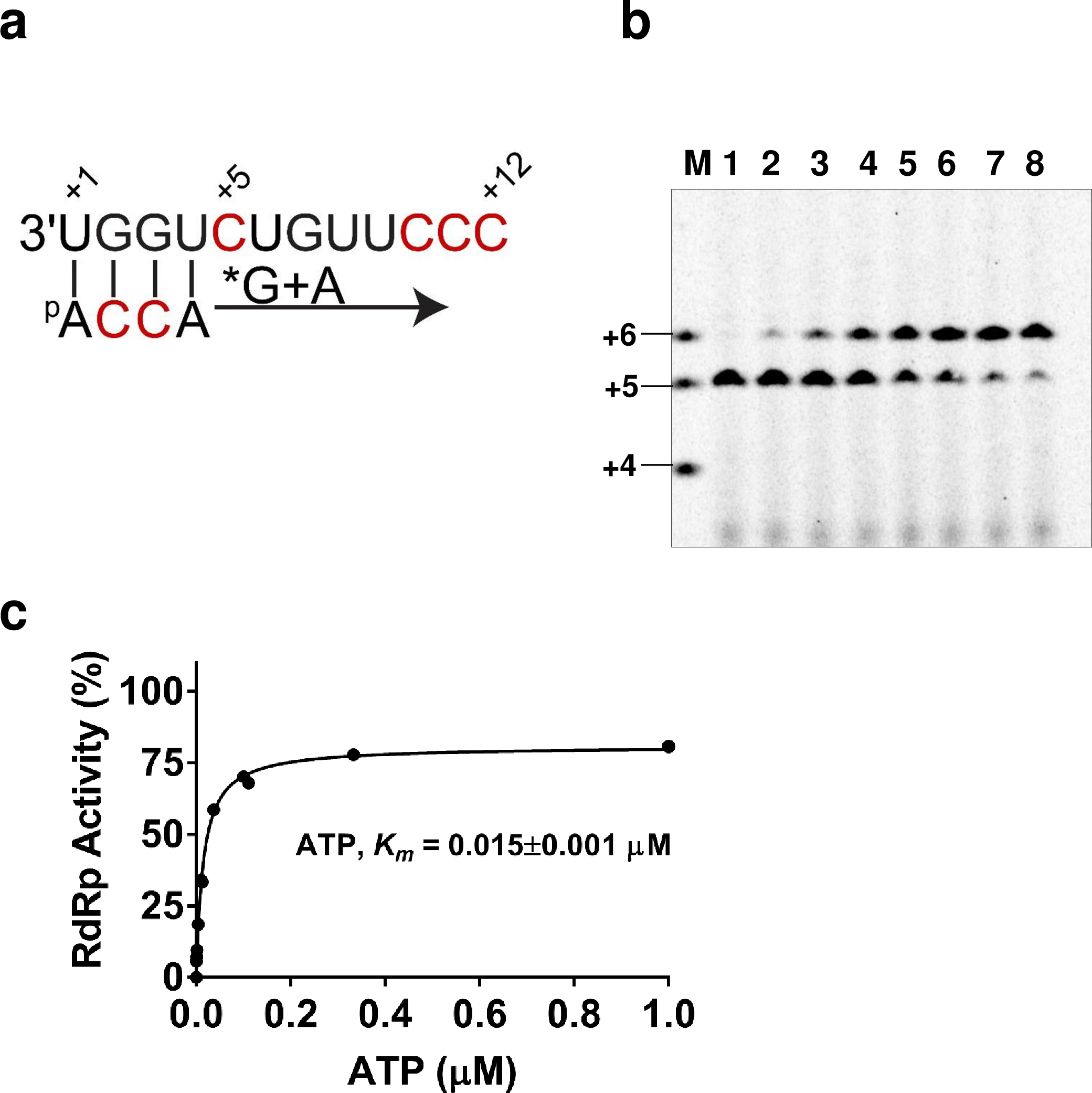

Supplement: S8 Fig — (a) A 12-mer template with a short 4-nucleotide prime was used to analyze the Km requirement for ATP. (b) A kinase-labeled primer was used as a marker to estimate size of extension products (lane M). NiV L(wt)-P, template, and primer were incubated in the presence of α33P-GTP tracer (lane 1), and with α33P-GTP combined with variable concentrations of ATP from 1nM to 1μM (lanes 2–8: 0, 0.001, 0.004, 0.01, 0.04, 0.01, 0.3, and 1μM ATP). (c) Data from multiple experiments was combined to generate a plot of RdRp Activity (%) vs ATP (μM), followed by a calculation of the Michaelis constant (Km). (TIF) [file ppat.1006889.s008.tif]

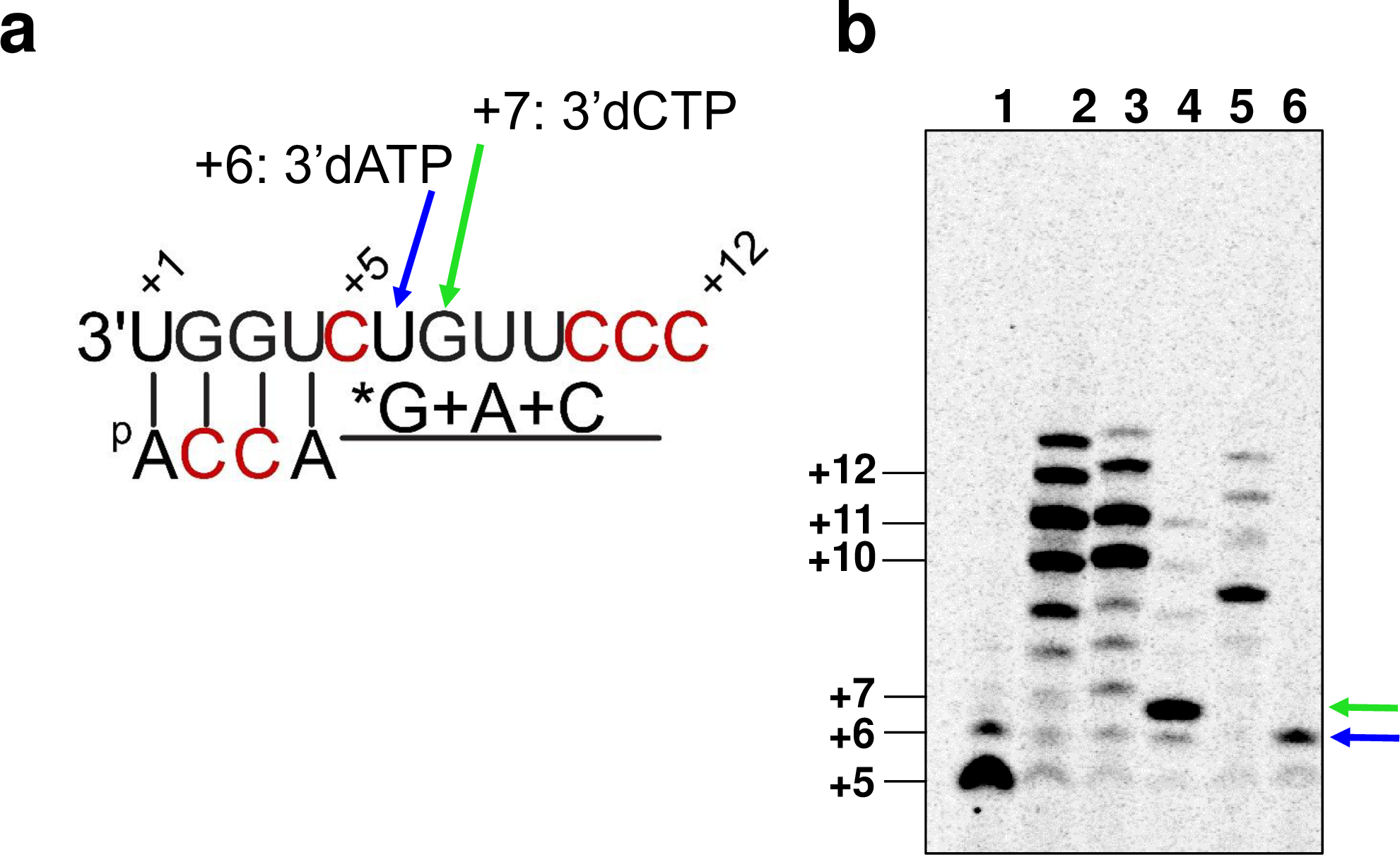

Supplement: S9 Fig — (a) A 12-mer template with four bases complementary to a short 4-nucleotide primer was used to analyze primer-extension activity of NiV L(wt)-P. Blue and green arrows indicate incorporation sites for 3′dATP or 3′dCTP at positions +6 or +7, respectively. (b) NiV L(wt)-P, template, and primer were incubated in the presence of α33P-GTP tracer (lane 1), α33P-GTP + ATP + CTP (lane 2), α33P-GTP + ATP + R1479-TP (lane 3), α33P-GTP + ATP + 3′dCTP (lane 4), α33P-GTP + CTP + GS-5734-TP (lane 5), and α33P-GTP + 3′dATP (lane 6). (TIF) [file ppat.1006889.s009.tif]

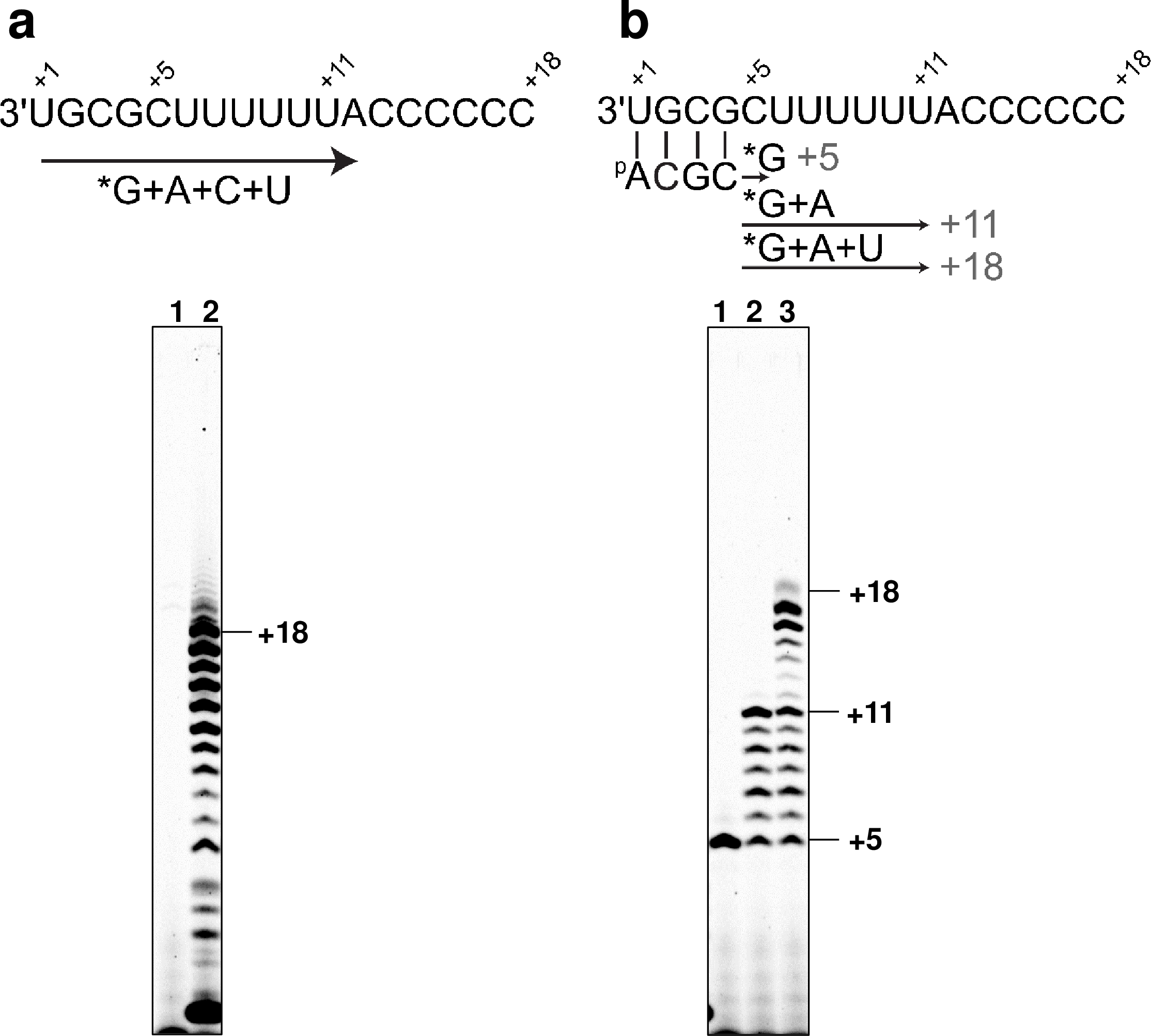

Supplement: S10 Fig — (a) RSV L-P, an 18-mer template, were incubated in the presence of α33P-GTP tracer (lane 1) or α33P-GTP + ATP + CTP (lane 2). (b) RSV L-P, an 18-mer template, and primer were incubated in the presence of α33P-GTP tracer (lane 1), α33P-GTP + ATP (lane 2), or α33P-GTP + ATP + UTP. (TIF) [file ppat.1006889.s010.tif]

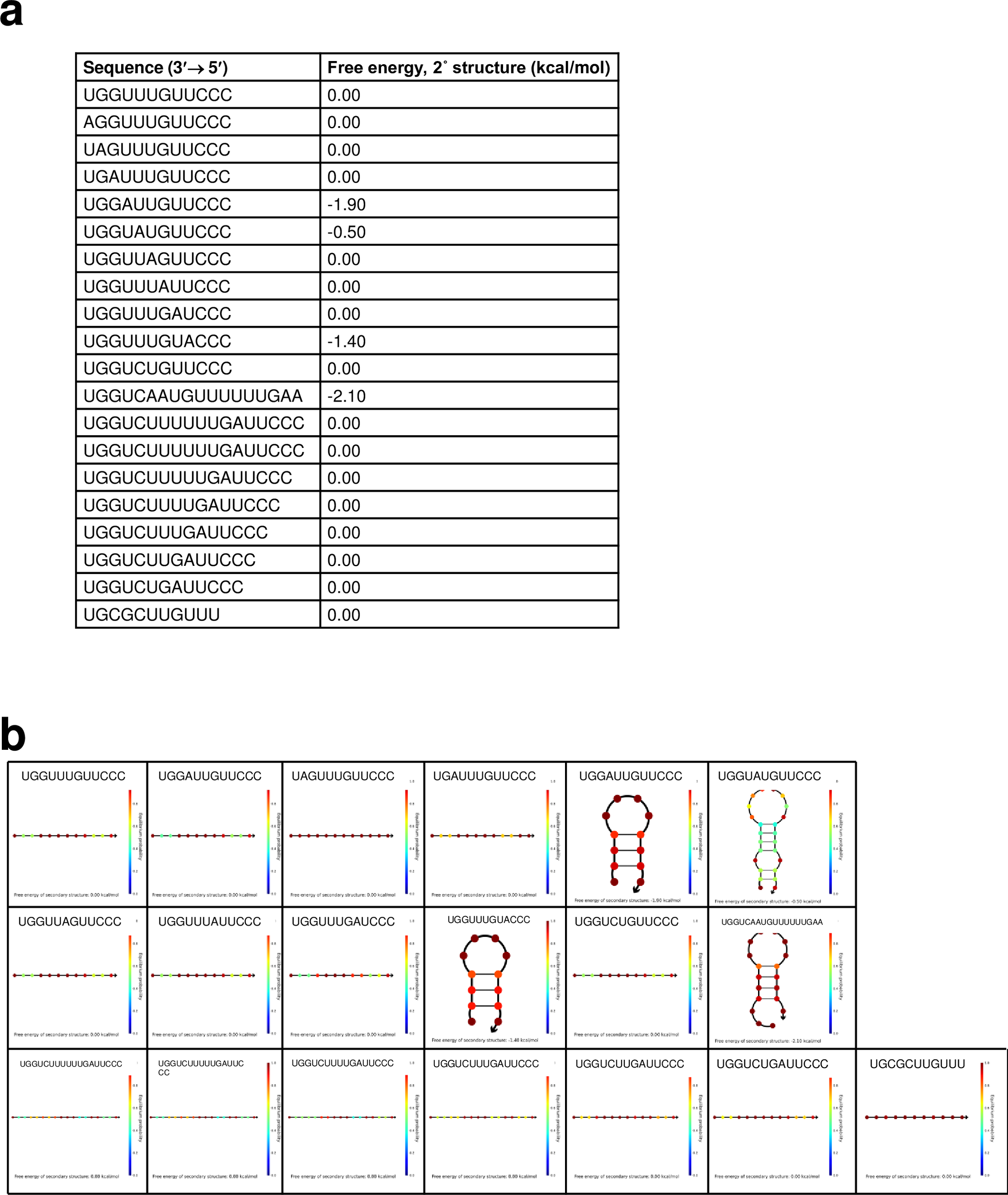

Supplement: S11 Fig — (a) The RNA templates used in RNA synthesis reactions with NiV with free energies associated with secondary structure formation (kcal/mol). (b) Predicted secondary structures for RNA. (TIF) [file ppat.1006889.s011.tif]
